# Calreticulin Shortage Results in Disturbance of Calcium Storage, Mitochondrial Disease, and Kidney Injury

**DOI:** 10.3390/cells11081329

**Published:** 2022-04-13

**Authors:** Asima Tayyeb, Gry H. Dihazi, Björn Tampe, Michael Zeisberg, Desiree Tampe, Samy Hakroush, Charlotte Bührig, Jenny Frese, Nazli Serin, Marwa Eltoweissy, Gerhard A. Müller, Hassan Dihazi

**Affiliations:** 1 School of Biological Sciences, University of the Punjab, Lahore 53700, Pakistan; asima.sbs@pu.edu.pkm; 2 UMG-Laboratories, Institute for Clinical Chemistry, University Medical Centre Göttingen, Robert-Koch-Strasse 40, 37075 Göttingen, Germany; gryhelene.dihazi@med.uni-goettingen.de; 3 Clinic for Nephrology and Rheumatology, University Medical Centre Göttingen, Robert-Koch-Strasse 40, 37075 Göttingen, Germany; bjoern.tampe@med.uni-goettingen.de (B.T.); michael.zeisberg@med.uni-goettingen.de (M.Z.); desiree.tampe@med.uni-goettingen.de (D.T.); charlotte.buehrig@gmx.de (C.B.); nazli.serin@med.uni-goettingen.de (N.S.); gmueller@med.uni-goettingen.de (G.A.M.); 4 Department of Pathology, University Medical Centre Göttingen, Robert-Koch-Strasse 40, 37075 Göttingen, Germany; samy.hakroush@googlemail.com; 5 Department of Occupational Medicine and Health Safety, Deutsche Post AG, Kölnische Strasse 81, 34117 Kassel, Germany; jenny.frese@med.uni-goettingen.de; 6 Department of Hematology and Oncology, University Medical Centre Göttingen, Robert-Koch-Strasse 40, 37075 Göttingen, Germany; 7 Department of Zoology, Faculty of Science, Alexandria University, Alexandria 21568, Egypt; marwaeltoweissy@alexu.edu.eg; 8 Centre for Biostructural Imaging of Neurodegeneration (BIN), University Medical Center Göttingen, 37075 Göttingen, Germany

**Keywords:** calreticulin, calcium signaling, mitochondrial disease, autophagy, oxidative stress, kidney fibrosis, calcium storage

## Abstract

Renal Ca^2+^ reabsorption plays a central role in the fine-tuning of whole-body Ca^2+^ homeostasis. Here, we identified calreticulin (Calr) as a missing link in Ca^2+^ handling in the kidney and showed that a shortage of Calr results in mitochondrial disease and kidney pathogenesis. We demonstrated that Calr^+/−^ mice displayed a chronic physiological low level of Calr and that this was associated with progressive renal injury manifested in glomerulosclerosis and tubulointerstitial damage. We found that Calr^+/−^ kidney cells suffer from a disturbance in functionally active calcium stores and decrease in Ca^2+^ storage capacity. Consequently, the kidney cells displayed an abnormal activation of Ca^2+^ signaling and NF-κB pathways, resulting in inflammation and wide progressive kidney injury. Interestingly, the disturbance in the Ca^2+^ homeostasis and signaling in Calr^+/−^ kidney mice cells triggered severe mitochondrial disease and aberrant mitophagy, resulting in a high level of oxidative stress and energy shortage. These findings provide novel mechanistic insight into the role of Calr in kidney calcium handling, function, and pathogenesis.

## 1. Introduction

Chronic kidney disease (CKD) is becoming a major public health problem worldwide, affecting 7.2% of the global adult population [1]. Despite how they start, most renal diseases eventually converge into common histopathological impairments such as glomerulosclerosis and tubulointerstitial fibrosis, leading to progressive functional deterioration of the renal system [2]. In the last few decades, the progression of the disease process has been well documented. Much interest has focused on investigating potential mechanisms to prevent or reverse the damage. However, the intracellular mechanisms responsible for renal disease initiation leading to complete damage are mostly not well understood. Accumulating evidence from a focus on the molecular and cellular mechanisms of CKD, including our previous studies, revealed a pathophysiologic involvement of endoplasmic reticulum (ER), especially ER Ca^2+^-binding proteins in renal disease progression [3,4,5,6,7]. Therefore, ER Ca^2+^-binding proteins have become an area of interest to understand the possible links in renal disease initiation and progression.

Calr is a ubiquitously expressed ER resident Ca^2+^-binding chaperon [8]. Biochemical and structural studies have demonstrated three distinct structural and functional domains of Calr: the amino-terminal lectin-binding N-domain for chaperone function of the protein; the middle proline-rich P-domain assisting in both Ca^2+^ storage and chaperone activity; and the carboxyl-terminal, highly acidic Ca^2+^-binding and -storing C-domain followed by an ER retention/retrieval signal on C-terminal [8,9,10]. Within ER, Calr plays two important functions: as a chaperon in quality control and as ER Ca^2+^-binding and -storage proteins. Consistent with this is a direct correlation of Calr expression with ER Ca^2+^ storage capacity [3,10,11,12,13]. In addition to storage of Ca^2+^, Calr is also known to modulate Ca^2+^ signaling and homeostasis through store-operated Ca^2+^ influx from plasma membrane. It interacts with Ca^2+^ entry and exit channels SERCA and IP3R and modulates Ca^2+^ influx by controlling the extent of inositol 1,4,5-trisphophate-induced Ca^2+^ store depletion [14,15,16].

Calr deficiency in mice is lethal and homozygote animals mostly die between E12 and E15 due to impaired heart development [17]. Recently, we demonstrated that Calr deficiency results in the disturbance of ribosome biogenesis and the retardation of embryonic kidney development [18]. Many extracellular functions of Calr have been reported, including roles in immunogenic cell death in cancer, cellular adhesion, cell migration, phagocytosis, inflammation, cell signaling, and enhancing wound healing [19]. Additionally, we have demonstrated that Calr plays an important role in the functioning and survival of renal cells under stress conditions [3].

In the present study, we investigated the impact of a chronic low level of Calr on kidney function and physiology in Calr^+/−^ mice. We demonstrated that Calr is a missing player in the calcium reabsorption in kidney, and that Calr^+/−^ mice suffer from an imbalance in Ca^2+^ homeostasis, an increased cytosolic Ca^2+^ concentration, resulting in the activation of Ca^2+^ signaling, oxidative stress, autophagy, and renal injury.

## 2. Materials and Methods

### 2.1. Animals

Calreticulin heterozygote (Calr^+/−)^ and wild type (Calr^+/+^) littermate mice in identical C57BL/6J genetic backgrounds were obtained from Prof. Marek Michalak, University of Alberta, Edmonton, AB, Canada. Mice were bred under specific pathogen-free housing conditions and genotyped as previously described in Mesaeli et al. [17]. Morphological and biochemical analyses of adult kidneys at three time points of an average age 15, 30, and 40 weeks (wk) were carried out. All experimental procedures were performed according to the German Animal Care and Ethics Legislation (NIH standards) and were approved by the local Ethics Committee of the University Medical Center Göttingen, Germany (33.14-42502-04-11/0598).

### 2.2. Morphometric and Histological Examinations of the Kidneys

For morphometric and histological analyses, ten mice of each age and genotype group (15, 30, and 40 weeks (wk)) were sacrificed and the kidneys were dissected from the animals. The freshly harvested kidneys were cleaned of surrounding fat, washed in sterile saline solution, and weighed. Kidneys were dissected along sagittal section for macroscopic and microscopic analyses of the renal injury in Calr^+/−^ mice. The macroscopic differences in Calr^+/−^ kidneys compared to Calr^+/+^ controls were recorded using a Nikon D5000 Camera (Nikon, Tokyo, Japan). Data were recorded from all the mice included in the study.

For histological analyses, freshly harvested kidneys were immediately fixed overnight in a freshly prepared 5% paraformaldehyde solution. Fixed kidneys were processed for paraffin embedding and sectioning using standard procedures. Further, 3 µm thick tissue sections were stained with various histological methods to illustrate potential pathological impairment in the kidney tissue of Calr^+/−^ animals. The following staining techniques were performed for light microscopic examination and histological evaluation: periodic acid–Schiff (PAS), hematoxylin–eosin (HE), Elastika van Gieson elastin staining according to Weigert, Gomori’s stain, reticulin stain, and Jones-HE staining. Histological analysis was performed with ImageJ software version 1.52 (http://imagej.nih.gov/ij/, accessed on 4 December 2018) as described by Rangan and Tesch [20]. Briefly, the mean glomerular areas (mGA) of at least 30 glomeruli tuft/animal group were measured. PAS-positive material in each of these glomeruli was quantified and expressed as the mean mesangial area (mMA).

### 2.3. Immunohistological Analysis of Kidneys

Immunostaining of deparaffinized and rehydrated sections was performed to detect the tissue distribution and expression of several proteins. Following antigen retrieval pretreatment in 0.01 M citric acid using a steamer for 25 min, the endogenous peroxidase was inactivated with 3% H_2_O_2_ in phosphate buffered saline (PBS) for 10 min at room temperature in the dark. The sections were blocked with 10% goat serum in PBS for 1 h and incubated with primary antibodies overnight at 4 °C. Primary antibodies were detected with Horseradish Peroxidase (HRP) labeled secondary antibody for 1 h at room temperature (GE Healthcare, Chicago, IL, USA). For negative controls, tissue sections were incubated only with the secondary antibody. The detection reaction was developed with 3,3-diaminobenzidine (Sigma, Taufkirchen, Germany) for 10 min at room temperature in the dark. Nuclei were counterstained with hematoxylin prior to examination. All tissue sections were dehydrated in graded alcohols and xylene and embedded in mounting solution Entellan (Merck, Darmstadt, Germany).

For fluorescence staining the primary antibodies were detected with fluorescence Alexa 555–conjugated goat anti-rabbit or Alexa 488–conjugated goat anti-mouse secondary antibody (Invitrogen, Waltham, MA, USA) as recommended. Slides were rinsed and mounted with Vectashield 4,6-diamidino-2-phenylindole (DAPI) (Vector Laboratories, Eching, Germany) to visualize the nuclei.

### 2.4. Electron Microscopy

For ultrastructural electron microscopy (4 mm), three kidney samples were taken from three mice per group and fixed in Karnovsky solution. After dehydration in a graded series of ethanol, tissue samples were cleared in propylene oxide and embedded in epoxy resin, as previously described [21]. Ultrathin sections (70 nm) were prepared (Reichert-Jung Ultracut E; Leica, Wetzlar, Germany) and examined under an electron microscope (LEO 906E; Zeiss, Oberkochen, Germany).

### 2.5. Laboratory Parameters and ELISA

All laboratory parameters (e.g., Ca^2+^, Cl^−^, Urea, Na^+^) were measured by standard routine methods in the accredited laboratory of the University Medical Center, Göttingen. To analyze the routine parameters, samples were collected from 30 mice per group (Calr^+/+^ and Calr^+/−^).

The concentrations of parathyroid hormone (PTH) and 1.25[(OH)_2_) vitamin D (VD3) in mouse serum were measured using mouse PTH ELISA (Uscn Life Science, Houston, TX, USA) and mouse VD3 ELISA (Enzo life Science, Lörrach Germany) kits. The kits have a minimum detection limit of 9.8 pg/mL and 1.98 ng/mL, respectively, and a measurable concentration range of 9.8–800 pg/mL and 0.5–1010 ng/mL, respectively. For this purpose, serum samples from 25 Calr^+/+^ and 25 Calr^+/−^ were collected and the samples were diluted 10-fold before measurement. The PTH and VD3 concentrations were corrected for dilutions.

### 2.6. Impact of Calcium Handling Hormones on Calr Expression

To investigate whether the Calr expression underlies the control of the calcium handling hormones, both MDCK cells and tubular mouse primary cells, isolated from Calr^+/+^ and Calr^+/−^ mouse kidney according to [22], were cultured to 70% confluency. The cells were incubated in FCS-free medium before treatment with PTH (1 nM) and VD3 (10 nM) for 48 h and subsequently harvested and lysed with the lysis buffer. The expression level of Calr and Grp78 was estimated using Western blot and antibodies against Calr and Grp78. Actin beta (Actb) was used as loading control. Data analyses and quantification were performed using the ImageJ software.

### 2.7. Calcium Investigations in Primary Tubule Cells

To investigate the impact of low Calr levels on free calcium in kidney tissue cells, intact perfused kidney slices from Calr^+/−^ and Calr^+/+^ were prepared and loaded with Rhod-2 to stain free calcium following the protocol of Crawford et al. [23].

We used Fura-2 as an indicator of intracellular calcium concentration. Fura-2 is a dual excitation ratiometric indicator; the Ca^2+^-free form of Fura-2 has a peak excitation wavelength of 380 nm, whereas the peak excitation wavelength for Ca^2+^-bound Fura-2 is 340 nm. An elevation of Ca^2+^ concentration induces an increase in Fura-2 emission fluorescence when the indicator is excited at 340 nm, with a corresponding decrease in fluorescence at 380 nm excitation. The ratio of Fura-2 emission intensities at 340 nm excitation versus 380 nm is proportional to Ca^2+^ concentration.

For the determination of cytosolic free calcium, tubular kidney primary cells from Calr^+/+^ and Calr^+/−^ were isolated according to Yang et al. [22] and cultured to 70% confluency. The cells were loaded for 30 min with cell permeable Fura-2/AM. A 90% volume exchange was achieved within 10 s. Fura-2 fluorescence was measured in single cells by means of a ratio fluorescence imaging system on an Olympus IX-71 epifluorescent microscope (Olympus, Tokyo, Japan), equipped with Polychrome V (TillPhotonics, Kaufbeuren, Germany) as light source and an iXon 850 (Andor, Wiesbaden, Germany) EMCCD camera. Chopper frequency and complete data acquisition were controlled by software for a microcomputer system (PTI). The Fura-2 fluorescence emission of the cell monolayer at the excitation wavelengths 340 and 380 nm was recorded with an EMCCD camera using a 510 nm wide band filter (Olympus). The autofluorescence of the cells and background fluorescence were measured and automatically subtracted from the calcium signals. Calibration of the Fura-2 fluorescence signal was performed in the presence of the calcium ionophore ionomycin (10 µmol/L).

### 2.8. Mouse Kidney Cell Sub-Proteomes Isolation and Mass Spectrometric Analyses

#### 2.8.1. Isolation of Mitochondria, Nuclear, and Cytosolic Fractions

Forty-week-old Calr^+/+^ and Calr^+/−^ mouse kidneys were isolated, harvested, and prepared for protein extraction. The separation of the samples into mitochondrial, cytosolic, and nuclear fractions was performed according to the protocol of an isolation kit for tissues and cultured cells (Amsbio, Abingdon, UK). In total, 150 mg of freshly harvested mouse kidneys was washed twice in 10 mL ice-cold PBS and then minced and homogenized with a Douncer homogenizer in 1 mL mitochondria isolation buffer supplied with protease inhibitors to avoid protein degradation. The suspension was centrifuged for 10 min at 600× *g* and 4 °C. The supernatant was transferred to a new tube and the pellet consisting of a nuclear fraction was resuspended in lysis buffer supplied with protease inhibitor. The tubes with the supernatant were centrifuged at 12,000× *g* for 15 min at 4 °C; the resulting supernatant is the cytosolic fraction. The pellet, which contained the mitochondrial fraction, was washed in mitochondrial isolation buffer by repeating the above centrifugation step. Isolated mitochondrial pellets were either resuspended in mitochondria storage buffer for intact mitochondria for functional assay or lysed with mitochondria lysis buffer for mass spectrometric or Western blot analyses.

#### 2.8.2. SDS-PAGE, In-Gel Tryptic Digestion, and Mass Spectrometric Analyses

The protein extracts from the different fractions were separated in polyacrylamide gel electrophoresis (SDS-PAGE). The gels were stained with Coomassie blue, and the visualized protein lanes were excised in 20-band sections and subjected to in-gel digestion with trypsin. After peptide extraction, the resulting tryptic digests were analyzed using the Thermo Scientific Q Exactive and the tandem mass spectra were extracted. All MS/MS samples were analyzed using Mascot (Matrix Science version 2.4.1., London, UK, accessed on 4 December 2018). Mascot was set up to search the SwissProt_2015_07 database (selected for Mus musculus, 16723 entries) with trypsin as the digestion enzyme. Mascot was searched with a fragment ion mass tolerance of 0.020 Da and a parent ion tolerance of 10.0 ppm. Carbamidomethyl of cysteine was specified in Mascot as a fixed modification and the oxidation of methionine was specified as a variable modification. Scaffold (version Scaffold_4.4.5, Proteome Software Inc., Portland, OR, USA, accessed on 4 December 2018) was used to validate MS/MS-based peptide and protein identifications. The software normalizes the MS/MS data between samples. This allowed us to compare the abundance of a protein between samples. For the normalization, the samples were prepared and processed under the same experimental conditions. Under such conditions, individual proteins may be up- or down-regulated, whereas the total amount of protein is the same in each sample. There are two levels of “sample” in Scaffold. The MS level is the sample run through the mass spectrometer, the second level is the biological sample, and we performed replicates in both levels. Frequently, the biological sample is fractionated into multiple MS samples. Scaffold allows us to view the MS samples within a biological sample or to combine all the MS samples into a single sample using the “MuDPIT” option. Normalization is carried out at the level of the MS sample. The normalization method that Scaffold uses is to sum up the “Unweighted Spectrum Counts” for each MS sample. These sums are then scaled so that they are all the same. The scaling factor for each sample is applied to each protein group and adjusts its “Unweighted Spectrum Count” to a normalized “Quantitative Value”.

Peptide identifications were accepted if they could be determined with a probability greater than 80.0% to achieve a False Discovery Rate (FDR) less than 1.0% by the Scaffold Local FDR algorithm. Protein identifications were accepted if they could achieve an FDR less than 1.0% with a probability greater than 28.0% and contained at least two identified peptides. Protein probabilities were assigned by the Protein Prophet algorithm [24]. Proteins containing similar peptides and which could not be differentiated based on MS/MS analysis alone were grouped to satisfy the principles of parsimony. Proteins sharing significant peptide evidence were grouped into clusters. Proteins were annotated with Gene Ontology (GO) terms from NCBI (accessed on 21 July 2015) and NCBI (accessed on 11 August 2015).

### 2.9. Protein Kinase A (PKA) and Protein Kinase C (PKC) Activity Assays

The PKA and PKC activities were investigated using the PKA kinase activity kit and PKC kinase activity kit (Enzo life Science, Lörrach Germany), respectively. The activity assay for PKC is an ELISA-based assay, which uses an antibody that recognizes and quantifies the amount of the specific phosphorylated peptide substrate of PKC. The PKC activity measurement was performed in kidney primary cells from Calr^+/+^ and Calr^+/−^, according to the manufacturer’s recommendation. The PKA activity was also measured using an ELISA kit designed for the PKA assay.

### 2.10. Cytochrome C Oxidase Activity Assay

Cytochrome c oxidase(Cox) activity was determined in intact isolated mitochondria from kidney tissues using the Cox kit according to the manufacturer’s instructions (Mitochondrial activity assay kit, Amsbio). The colorimetric assay is based on the observation that a decrease in the absorbance of ferrocytochrome c at 550 nm is caused by its oxidation to ferricytochrome c by Cox.

### 2.11. Bioinformatic Analyses

To examine potential protein function categories and pathways of significantly regulated proteins, we performed bioinformatics analysis using the following publicly available protein software: DAVID Functional Annotation Bioinformatics Microarray Analysis David Bioinformatics version 6.8, (http://david.abcc.ncifcrf.gov/, accessed on 23 October 2016), the protein–protein interaction network software String 10.5 (https://string-db.org, accessed on 1 October 2016), and the pathway analysis software Kegg: Kyoto Encyclopedia of Genes and Genomes version 80.0 (https://www.genome.jp/kegg/, accessed on 1 October 2016).

### 2.12. Western Blot Analysis

Western blot analyses were performed according to Towbin et al. [25]. Equal amounts of proteins (50–75 µg) were separated by SDS-PAGE and transferred on nitrocellulose membranes (Amersham Pharmacia Biotech, Buckinghamshire, UK). The membranes were blocked in 5% non-fat dry milk in a TBST buffer (20 mM Tris-HCl, pH 7.4, 150 mM, NaCl 0.1%, Tween 20) and incubated with the indicated primary antibody at 4 °C overnight. To visualize the protein bands, fluorescence labeled secondary antibodies were used. To confirm equal protein loading, the blots were treated with anti-*β*-actin antibody (Sigma, Taufkirchen, Germany).

### 2.13. Data Analysis

All blots were quantified using the ImageJ software. GraphPad Prism version 8.0.1., GraphPad (https://www.graphpad.com/, accessed on 4 December 2018) was used for graphical presentation and analysis by either Student’s *t*-distribution or one-way ANOVA. The results are presented as the mean ± SD of at least three biological replicates and from each of at least three independent experiments. Differences were considered statistically significant when *p* < 0.05.

### 2.14. Antibodies

Polyclonal rabbit anti-Fn1 (F3648), anti-Lamc1 (L9393), anti-Grp78 (G8918), anti-Park7 (HPA004190), and mouse monoclonal anti-Actb (A5441) antibodies were from Sigma (Taufkirchen, Germany). Polyclonal rabbit anti-Ddit3/Chop (PAB18326) and anti-Sod1 (PAB18299) antibodies were from Abnova. Polyclonal rabbit anti-LC3 (NB-100-2220) was from Novus Biologicals (Centennial, CO, USA); rabbit monoclonal anti-Becn-1 (11//2016) was from Cell Signaling (Frankfurt am Main, Germany); rabbit monoclonal anti-Eno1 (ab155102), anti-Prdx6 (EPR3754), anti-iNos1 (ab178945), anti-Ncx1 (ab177952), anti-Pvalb (ab181086), anti-Phb (ab75766), anti-p65 (ab32536), anti-Trpv5 (ab137028), anti-Vdac1 (ab154856), anti-Col-1 (ab270994), rabbit polyclonal anti-Bcl-2 (ab196495), goat anti-Calr (ab2907), anti-Pdia3 (ab228789), anti-pEif2-alpha (ab131505), mouse monoclonal anti-Calm1 (ab2860), anti-Canx (ab112995), and anti-IkB (ab12134) antibodies were from Abcam (Berlin, Germany). Mouse monoclonal anti-Cyc antibody (37BA11) was from Agilent Dako (Santa Clara, CA, USA).

## 3. Results

### 3.1. Chronic Low Level of Calr Results in Kidney Injury

#### 3.1.1. Low Level of Calr Results in Progressive Damage in Calr^+/−^ Mouse Kidney

Calr gene knockout is embryonic lethal and Calr^−/−^ embryos die during embryonic stages with multiple developmental defects, especially heart development [17].

**Figure 1:**
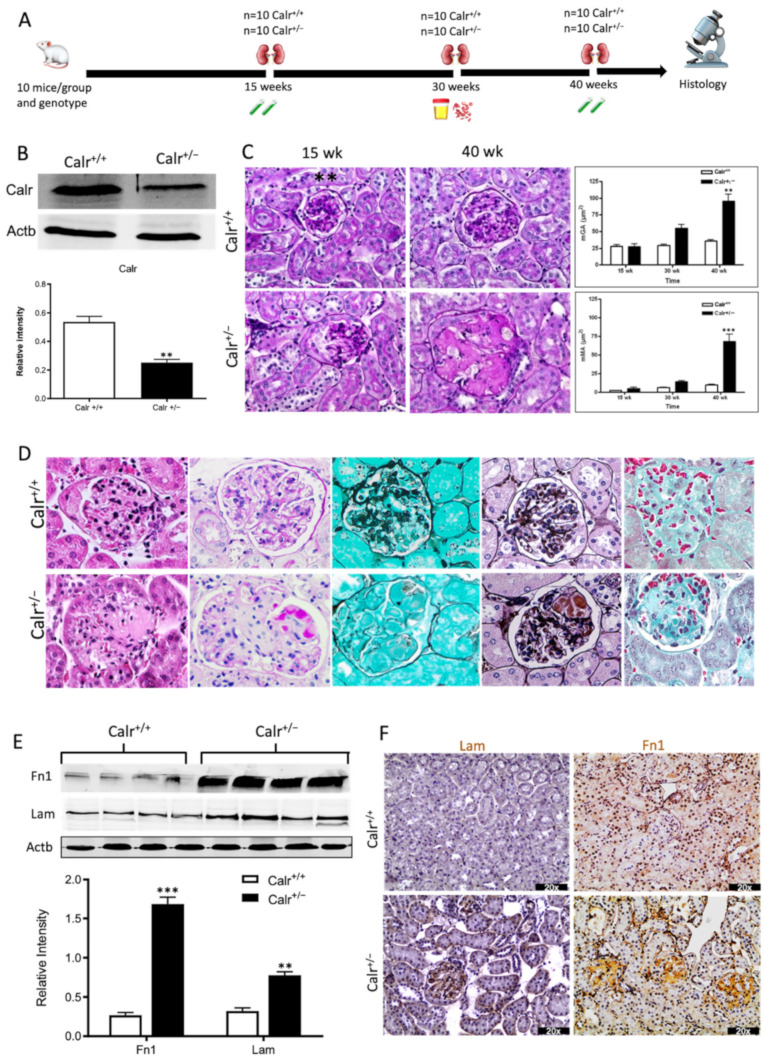

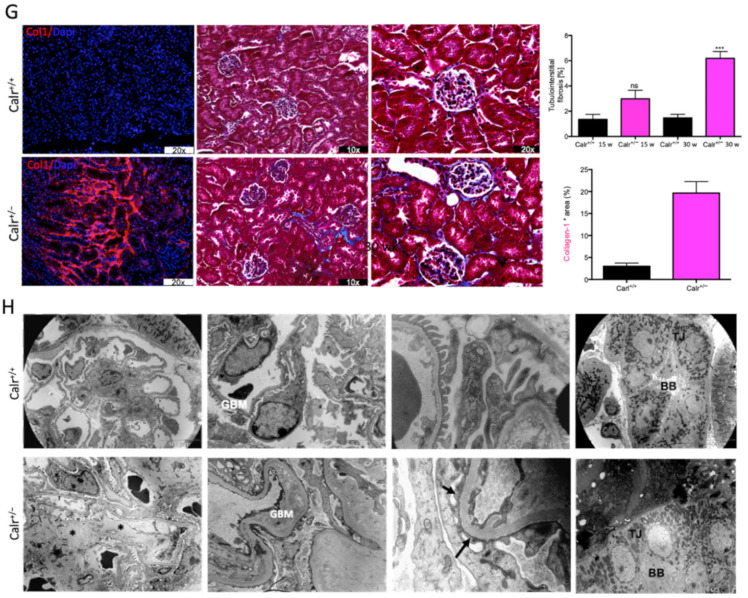
Progressive structural alterations in Calr^+/−^ mice. (**A**): Illustration of the experimental design for structural investigation of the kidney. (**B**): Western blot analysis of Calr abundance in Calr^+/+^ and Calr^+/−^ mouse kidney showing a > 50% reduction in Calr expression in Calr^+/−^ mouse kidney. Paraffin-embedded kidney sections (3 µm) were stained with PAS to compare the kidney structures of Calr^+/+^ and Calr^+/−^ at 15 wk, 30 wk, and 40 wk of age (**: *p* < 0.01). (**C**): Representative glomeruli of PAS-stained sections from Calr^+/+^ and Calr^+/−^ mice (magnification ×40); upper bar diagram shows an increase in mGA in the kidneys of 30 and 40 wk old Calr^+/−^ mice in comparison to those of young Calr^+/−^ and Calr^+/+^ mice of the same age (*p* < 0.05). Lower bar diagram shows a significant increase in mMA in 40 wk old Calr^+/−^ mice compared to young Calr^+/−^ mice of 15 and 30 wk old and Calr^+/+^ mice of same age. The data shown are mean ± SE (*n* = 30 glomeruli per group, *p* < 0.05). PAS: periodic acid–Schiff; mGA: mean glomerular area; mMA: mean mesangial area. (**D**): Kidney section from 40 wk old mouse kidney stained with different methods confirming advanced kidney injury in Calr^+/−^ mice. (**E**): Western blot analysis of Fin and Lam showed a high expression in Calr^+/−^ mouse kidney compared to Calr^+/+^. Bar diagram represents the quantification of the Western blot results shown in (**E**), (*n* = 4. ***: *p* < 0.001, ns: non-significant, *p* > 0.05). *β*-actin (Actb) was used as loading control. (**F**): Representative images of kidney section from Calr^+/+^ and Calr^+/−^ mice, staining of the kidney sections with antibodies against fibronectin (Fin1), and laminin. (**G**): Immunofluorescence staining with antibody against collagen I, MTC staining of kidney sections from Calr^+/+^ and Calr^+/−^ mice. The staining showed a significant marked increase in fibronectin, laminin, and collagen expression in the Calr^+/−^ kidney. (**H**): Kidney sections from 40 wk old Calr^+/+^ and Calr^+/−^ mice were assessed by electron microscopy. GBM: glomerular basement membrane; BB: brush borders; TJ: tight junction.

Our previous studies [3,18,26] revealed that an alteration in Calr expression significantly impacts renal cell function, resistance to stress, and kidney embryonic development. To investigate the potential role of Calr in the pathomechanism of kidney diseases, Calr knockout mice were used. Calr^+/−^ mice apparently live a normal life and present no detectable phenotype [17]. However, adult Calr^+/−^ mice express a chronic low level of Calr [17]. Investigations of Calr expression in kidney confirmed the low level of the protein in Calr^+/−^ adult mouse kidney (Figure 1A), and the heterozygous kidney expresses 50% less Calr than the wild type (Figure 1B). Kidneys from 10 animals and three age groups (15, 30, and 40 weeks) each were excised and prepared for morphological and histological investigations (Figure 1A, Supplementary Materials Figure S1A). The kidneys from the Calr^+/−^ mice showed age-dependent progressive deterioration with hypertrophic organs in some cases (Supplementary Materials Figure S1B). When observing over 500 animals, we found out that more than 50% of Calr^+/−^ old animals (40 ± 5 wk) showed severely affected kidneys with significant morphological differences compared to young Calr^+/−^ (15 ± 2 wk) and Calr^+/+^ controls of the same age (Supplementary Materials Figure S1C). About 1.1% (six animals) of the total examined Calr^+/−^ mice (> 560 animals) displayed hemizygous kidneys with one missing kidney.

#### 3.1.2. Calr^+/−^ Mice Develop Progressive Glomerulosclerosis and Tubulointerstitial Damage

The morphological aberrations reveal impaired kidney tissue structures of Calr^+/−^ mice. To investigate the renal tissue structure and to confirm potential alterations, tissue kidney sections (3 µm) from 15, 30, and 40 wk old Calr^+/+^ and Calr^+/−^ mice were generated and stained with different staining techniques. The histological examination showed pathological alteration of the renal tissue of Calr^+/−^ mice. The tissue injuries were more prominent in older animals and affected both kidney structures: glomerular tufts and interstitial tubular part (Figure 1C,D, Supplementary Materials Figure S1D,E). Younger Calr^+/−^ mice (15 wk) did not show any significant alteration in kidney tissues when compared to the Calr^+/+^ mice (Figure 1C, Supplementary Materials Figure S1E). Histological screening of the group of Calr^+/−^ mice at the age of 30 wk revealed prominent glomerular lesions with clear mesangial expansion and apparently increased matrix deposition in the majority of the investigated animals (>50%) (Supplementary Materials Figure S1E). Kidney sections from 40 wk old Calr^+/−^ animals showed advanced glomerular damage characterized by sclerotic lesions (Figure 1C,D). In addition to glomerulosclerosis, the tubulointerstitial area, especially the distal convoluted tubule, was severely affected. There were a significant number of dilated, atrophic, and necrotic tubules with an expanded lumen (Supplementary Materials Figure S1D,E). Measurement of mean glomerular area and volume as a parameter for overall glomerular architecture showed a progressive increase in the glomerular size in animals from 15 to 40 wk (Figure 1C). Similar results were also observed when measuring the mean mesangial area. Moreover, at the advanced stage (40 wk), expansion in the mesangial matrix was manifested with a significant increase in the PAS-positive area (Figure 1D). The histological impairments were confirmed with different tissue staining methods including HE, Jones-HE, Gomori, and Gomori trichrom staining (Figure 1D).

#### 3.1.3. Calr^+/−^ Mouse Kidney Suffer from Increased Extracellular Matrix Deposition, Indicating a Progressive Renal Interstitial Fibrosis

Western blot analysis showed a significant increase in the abundance of renal injury markers fibronectin and laminin in the kidney of Calr^+/−^ mice compared to Calr^+/+^ (Figure 1E). Moreover, immuno-histochemical and immunofluorescence analyses demonstrated an enhanced expression of extracellular matrix (ECM) proteins in both the glomeruli and the tubulointerstitial parts of Calr^+/−^ mice. A strong deposition of fibronectin was observed in the mesangium of the glomeruli and interstitial areas of Calr^+/−^ compared to Calr^+/+^ (Figure 1F). Similar results were observed for laminin (Figure 1F). Immunofluorescence staining of Collagen I further confirmed the deposition of ECM proteins in the interstitial space of the Calr^+/−^ kidney (Figure 1G). Moreover, Masson trichrome staining and quantification of the level of interstitial fibrosis confirmed the increase in ECM expression and deposition as well as the onset and progression of fibrosis in the Calr^+/−^ kidneys (Figure 1G).

#### 3.1.4. Ultrastructural Analysis Shows Glomerular and Tubular Cell Damage in Calr^+/−^ Mice

The electron microscopy data confirmed the Western blot and immuno-histochemistry data. Ultrastructural analysis illustrated advanced renal injury in Calr^+/−^ mice (30 and 40 wk) compared to age-matched Calr^+/+^ mice (Figure 1H) and to young Calr^+/−^ mice kidneys (15 wk) (Supplementary Materials Figure S1F). Ultrastructural changes in Calr^+/−^ mice were characterized by a significant mesangial sclerosis, marked and irregular thickening of the glomerular basement membrane, and podocytes with foot process broadening and effacement. In addition to glomerular abnormalities, damages to the renal tubules, especially in distal convoluted tubules, were noted as a focal loss of the brush border of the epithelial lining of proximal renal tubules and the disturbance of tight junctions.

#### 3.1.5. Calr^+/−^ Mouse Kidneys Showed No ER Stress but Disturbance in the Expression of Ca^2+^-Binding Proteins

**Figure 2:**
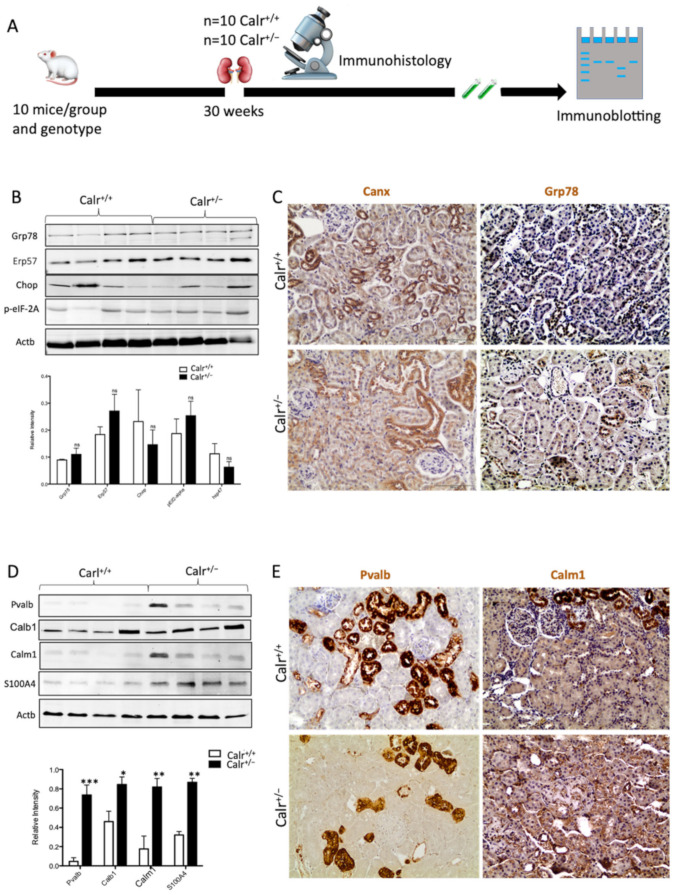
Impact of low level of Calr in the Calr^+/−^ mouse kidney on the expression of ER stress markers and EF-hand Ca2^+^-binding proteins. (**A**): Illustration of the experimental design for immunohistology and Western blot. (**B**,**C**): Western blot analyses and immunohistostaining of the ER stress markers Grp78, Erp57, Canx, Chop, and p-eIF-2A showed no significant difference between the Calr^+/+^ and Calr^+/−^ mouse kidneys; Actb was used as loading control. Below: Bar diagram represents the quantification of the Western blot results shown in (**B**) (*n* = 4. *: *p* < 0.05). (**D**,**E**): Western blot analyses and immunohistostaining of some ER-hand Ca^2+^-binding proteins Pvalb, Calb1, Calm1, and S100A4 showed significant up-regulation in Calr^+/−^ kidney compared to Calr^+/+^. Below: Bar diagram represents the quantification of the Western blot results shown in (**D**) (*n* = 4. *: *p* < 0.05, **: *p* < 0.01, ***: *p* < 0.001). The immunohistochemical staining of Grp78, Pvalb, Calm1, and Canx is shown in 40 wk old Calr^+/−^ and Calr^+/+^ mouse kidney sections.

Calr is a chaperone protein implicated in protein folding and in Ca^2+^ binding. The last function is responsible for Ca^2+^ storage and Ca^2+^ homeostasis regulation [8,10]. To determine which functional aspect of Calr was responsible for kidney injury in Calr^+/−^ mice, we investigated the expression of key proteins in the ER stress response as well as the proteins known to be involved in the Ca^2+^ binding (Figure 2A). Western blot analysis of the ER stress-induced proteins Grp78, Canx, and Erp57 revealed no effect of the low level of Calr on their abundance (Figure 2B,C). Moreover, the expression of two unfolded protein response (UPR) key proteins, Chop and p-eIF-2A, was not impaired in the Calr^+/−^ kidney (Figure 2B). These data ruled out the ER stress and the UPR pathway as operating aspects in renal injury in Calr^+/−^ mouse kidneys.

Disturbance in Ca^2+^ homeostasis and alteration in the free intracellular Ca^2+^ concentration frequently result in the differential expression of a group of EF-hand cytosolic Ca^2+^-binding proteins. Therefore, we examined the abundance of selected EF-hand Ca^2+^-binding proteins, including parvalbumin (Pvalb) and calmodulin (Calm1), by immunohistochemistry, and we examined Pvalb, Cam, S100A4, and calbindin (Calb1) by immunoblotting in the Calr^+/−^ and Calr^+/+^ kidneys. The Western blot data demonstrated a significant up-regulation of all investigated cytoplasmic EF-hand proteins (Figure 2D). Specific staining of distal convoluted tubules with Pvalb showed tubular damage in terms of a decrease in tubular lumen and damaged tubular walls (Figure 2E). The abundance and distribution of Calm1 were disturbed in Calr^+/−^, with overall nonspecific staining compared to high and specific staining of the distal convoluted tubule in Calr^+/+^ mice (Figure 2E). The data on EF-hand cytosolic proteins demonstrated that the impact of Calr level on ER-luminal Ca^2+^ affected the expression of EF-hand proteins, suggesting a role for Calr and ER calcium storage in the renal injury in Calr^+/−^ mice.

### 3.2. Effect of Low Level of Calr on Ca Homeostasis

#### 3.2.1. Alteration in the Calcium Homeostasis in the Calr^+/−^ Kidney

The up-regulation of cytoplasmic EF-hand Ca^2+^-binding proteins suggested an alteration in cytosolic Ca^2+^ level and likely in cellular Ca^2+^ balance. To investigate whether the chronic physiological low level of cellular Calr impacted the concentration of free Ca^2+^ in serum and in disturbance of Ca^2+^ reabsorption, we estimated the Ca^2+^ level in serum and urine of Calr^+/−^ and wild-type mice (Supplementary Materials Figure S2A). The free Ca^2+^ concentration in serum was significantly higher in Calr^+/−^ mice. Moreover, the alteration in Ca^2+^ concentration in Calr^+/−^ was more prominent in old mice compared to the young ones (Supplementary Materials Figure S2B(I)). The estimation of urine Ca^2+^ showed a decrease in the excretion in Calr^+/−^ mice, but this was statistically not significant compared to Calr^+/+^ animals (Supplementary Materials Figure S2B(II)). Investigation of sodium and chloride urinary concentration did not show any significant differences between the Calr^+/−^ and Calr^+/+^ mice (Supplementary Materials Figure S2C(I–II)), suggesting that the alteration in Ca^2+^ concentration had no effect on the body Na^+^ and Cl^−^ ions.

PTH and VD3 are known to play an important role in Ca^2+^ homeostasis. PTH is known to promote Ca^2+^ reabsorption in the kidney and increases the Ca^2+^ concentration in blood. ELISA investigation of the PTH and VD3 concentration showed a significant decrease in the level of both hormones in the serum of Calr^+/−^ animals when compared to Calr^+/+^ mice (Supplementary Materials Figure S2D(I–II)), which was in accordance with the increased serum Ca^2+^ concentration.

#### 3.2.2. Calreticulin Expression Is Subjected to PTH and VD3 Controls

Ca^2+^ imaging revealed a significantly higher level of free Ca^2+^ in Calr^+/−^ kidney cells (Supplementary Materials Figure S2E). Intact perfused kidney slices from Calr^+/−^ and wild type were loaded with Rhod-2 to stain free Ca^2+^, following the protocol of Crawford et al. [23]. Consistent with the primary cell data, the level of free Ca^2+^ was significantly higher in Calr^+/−^ mice tissues (Supplementary Materials Figure S2F). Moreover, we knocked down the Calr in MDCK cells using the CRISPR/cas9 system to achieve an in vitro system for Calr deficiency. Both qPCR and Western blot analyses demonstrated a significant down-regulation of Calr in the MDCK Calr-deficient cells compared to wild-type controls (Supplementary Materials Figure S2G,H). To investigate whether the MDCK Calr deficient cells had an impaired ER Ca^2+^ store, cells were treated with ATP and the Ca^2+^ ionophore A23187, followed by imaging of free cytosolic Ca^2+^ using Fura-2. Interestingly, the MDCK control cells released a high level of Ca^2+^ to cytosol upon treatment with ATP or A23187, whereas the MDCK Calr-deficient cells did not release Ca^2+^, revealing an impairment in ER Ca^2+^ store (Supplementary Materials Figure S2K).

**Figure 3:**
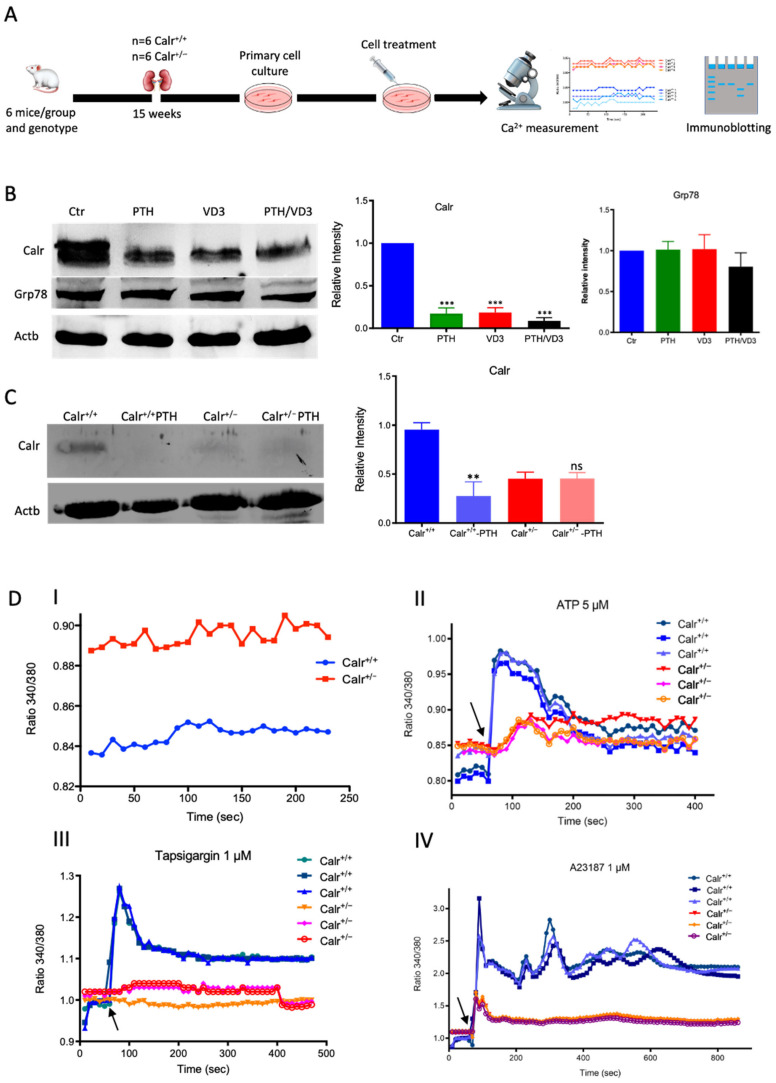

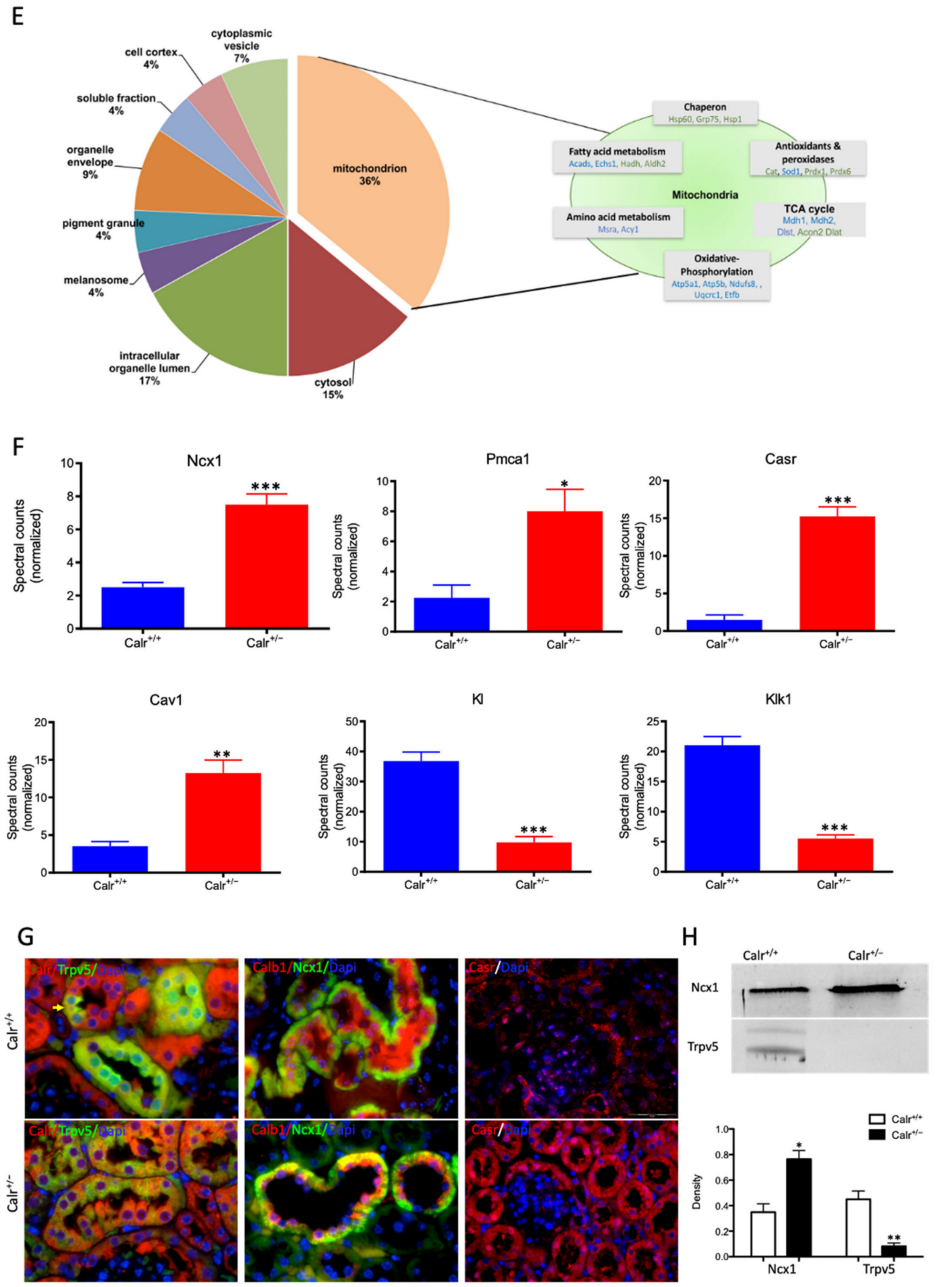
Calr expression underlays the control of calcium handling hormones and is important for Ca^2+^ homeostasis. (**A**): Illustration of the experimental design for calcium measurement and immunoblotting. (**B**): MDCK cells were treated with PTH or VD3, or both, and Calr expression was monitored using Western blot analysis. Compared to other ER stress markers, the expression of Calr was significantly down-regulated upon PTH or VD3 treatment. (**C**): Primary cell isolated from mouse kidneys (Calr^+/+^ and Calr^+/−^) were treated with PTH and the Calr expression was monitored using Western blot. Calr was significantly down-regulated upon PTH treatment. (**D**): The ratio of Fura-2 fluorescence emission intensity in response to 340 nm and 380 nm excitation (340/380) is proportional to intracellular (Ca^2+^). Fura-2 340/380 emission ratios are plotted against measurement time. (**I**): Average of Fura-2 340/380 emission ratios of Calr^+/+^ kidney primary cells (*n* = 20) and Calr^+/−^ kidney primary cells are plotted against time. Fura-2 340/380 emission ratios of three representative tubular kidney primary cells from Calr^+/+^ or Calr^+/−^ mice treated with ATP (5 µM) (**II**), Thapsigargin (1 µM) (**III**), or the ionophore A23187 (1 µM) (**IV**). (**E**): Categorization of differentially regulated proteins (2D gel analysis) was achieved by correlating GO identification numbers corresponding to cellular component and biological process with the regulated proteins. Values in figures presented the ratio distribution of proteins found in that respective category, (left) identified proteins categorized based upon their cellular component, (right) identified mitochondrial proteins categorized based upon their functional category. (**F**): Quantification of the proteomics data showed an up-regulation of protein sensing and transporting the calcium into the kidney cells. (**G**): Immunofluorescence staining of kidney tissue from Calr^+/+^ and Calr^+/−^ with antibodies against Trpv5, Calb1, Ncx1, and Casr. (**H**): Expression alteration of proteins involved in the regulation of calcium, Western blot analyses confirming the up-regulation of Ncx1 and the down-regulation of Trpv5. *: *p* < 0.05, **: *p* < 0.01, ***: *p* < 0.001.

The MDCK cell line was treated with either PTH or VD3, or a combination of both, and the expression of Calr was monitored (Figure 3A). Interestingly, all three treatments resulted in a significant decrease in Calr expression in MDCK cells (Figure 3B). Meanwhile, the treatments had no effect on other ER stress proteins such as Grp78. Primary tubule kidney cells (from Calr^+/+^ type and Calr^+/−^ mice) were treated with PTH and the expression of Calr was tested using Western blot. In the case of the primary cells from Calr^+/−^ kidney, the PTH did not significantly affect the Calr expression (Figure 3C). The results support the suggestion that in Calr^+/−^ kidney cells the level of Calr is 50% lower than in Calr^+/+^, which results in an alteration in storage capacity and an increase in the level of cellular free Ca^2+^. Moreover, the data clearly confirm that Calr expression is under the control of Ca^2+^ regulation hormones.

#### 3.2.3. Enhanced Cytosolic Free Ca^2+^ in Calr^+/−^ under Resting Conditions

To further demonstrate the disturbance in Ca^2+^ storage, we investigated whether Calr^+/−^ compared to Calr^+/+^ cells have an increased cytosolic free Ca^2+^ concentration and whether the ER Ca^2+^-store was disturbed. For this purpose, we first measured the cytosolic Ca^2+^ at rest in both Calr^+/−^ and Calr^+/+^ kidney primary cells by alternately exciting the Ca^2+^ indicator at 340 and 380 nm and recording emission fluorescence at 510 nm. In resting conditions, the fluorescence ratio showed a significant difference between Calr^+/−^ and Calr^+/+^ kidney cells independent of the cell preparation when we analyzed all experiments (Figure 3D,I). A significant alteration in cytosolic Ca^2+^ concentration was measured in Calr^+/−^ cells, which showed a higher concentration compared to Calr^+/+^ cells (Calr^+/+^ *n* = 20; Calr^+/−^ *n* = 21) (Figure 3D,I).

#### 3.2.4. Calr^+/−^ Kidney Cells Show Disturbance in Calcium Storage

The Ca^2+^ analysis in primary kidney cells from Calr^+/−^ and Calr^+/+^ under resting conditions revealed a disturbance in functionally active Ca^2+^ stores by Calr^+/−^ mice. To test this, we loaded Calr^+/−^ and Calr^+/+^ kidney tubule primary cells with the fluorescent cytosolic Ca^2+^ dye Fura-2 and measured the response upon addition of the Ca^2+^-ionophore A23187, thapsigargin, or ATP. ATP is known to induce Ca^2+^ release from IP3-sensitive stores after binding to purinergic receptors and the following activation of G-proteins and phospholipase C [27]. In our experiments, ATP induced an increase in free Ca^2+^ but only in Calr^+/+^ cells, whereas the Calr^+/−^ cells did not show any significant Ca^2+^ increase (Figure 3D(II)), indicating that Calr^+/−^ cells indeed did not possess sufficient IP3-sensitive stores that could be activated by ATP. Thapsigargin is a cell permeable inhibitor of the ER Ca^2+^ pump SERCA. It represses the Ca^2+^ uptake into the ER and results in raises in cytosolic Ca^2+^ concentration. As expected, thapsigargin-treated Calr^+/+^ kidney cells responded with a mobilization of the Ca^2+^ store and by increasing the concentration of cytosolic free Ca^2+^. In contrast, Calr^+/−^ cells did not show any significant Ca^2+^ elevation but maintained a low Ca^2+^ level similar to baseline (Figure 3D(III)). A23187 acts as a divalent cation ionophore and can transport Ca^2+^ across the cell membranes. A23187 is used to increase the intracellular Ca^2+^ level by depleting the ER Ca^2+^ store, allowing Ca^2+^ entry from extracellular space. In Calr^+/+^ kidney cells, the intracellular free Ca^2+^ concentration rapidly increased within 1 min after the initial stimulation with A23187 (Figure 3D(IV)). In contrast, Calr^+/−^ cells showed only a slight increase in Ca^2+^, confirming a reduced release of Ca^2+^ from the ER and supporting the data from ATP and thapsigargin concerning the disturbance in Ca^2+^ storage capacity.

### 3.3. Comparative Proteomic Analyses Showed Significant Alteration in the Proteome and Revealed a Severe Metabolic Disregulation in the Calr^+/−^ Mouse Kidneys

The histological analyses and the investigations of Ca^2+^ regulation showed that the chronic low level of Calr is inconvenient for Ca^2+^ balance and normal kidney function. To further investigate the link between disturbance in Ca^2+^ balance and kidney injury in Calr^+/−^ mouse kidneys, wide proteomic analyses were carried out on kidney tissue extracts using two approaches (Supplementary Materials Figure S3A). The first approach was 2D gel-based comparative proteome analysis of kidney protein extract obtained from 40 wk old Calr^+/+^ and Calr^+/−^ mice. The 2D pattern showed significant alteration in Calr^+/−^ kidney proteome (*p* < 0.05). The identification of the differentially abundant proteins revealed an alteration in the oxidative stress pathway and in oxidative phosphorylation but ruled out ER stress involvement in kidney injury in Calr^+/−^. ER stress markers such as Grp78 and Erp57 were not regulated (Supplementary Materials Figure S3B,C). A large part of the regulated proteins was identified as mitochondrial proteins (Figure 3E). Interestingly, oxidative stress proteins were up-regulated, indicating increased ROS in Calr^+/−^ kidney. In contrast to the other oxidative stress marker, Sod1 was down-regulated in Calr^+/−^ samples (Supplementary Materials Figure S3C). To better explore the proteome alteration in Calr^+/−^ kidney, a fractionation step followed by deep proteome investigation were carried out. We generated four protein fractions (mitochondrial, membrane, cytosolic, and nuclear fractions) from each kidney sample (Calr^+/+^ and Calr^+/−^). Subsequently, the procedure including 1D-SDS-PAGE separation followed by in-gel digestion and LC-MS/MS with spectral account quantification was carried out for each sample (Supplementary Materials Figure S4A). To get an overview on the expression changes in the proteome between Calr^+/+^ and Calr^+/−^ kidneys in the large set of data obtained from the proteomic analyses, volcano plots were generated by plotting the fold-changes versus significance. This allowed quick identification of proteins that were statistically significant and differentially expressed between the Calr^+/+^ and Calr^+/−^ kidneys (Supplementary Materials Figure S4BI–IV). A pie chart comparative presentation of the mass spectrometry data allowed an overview of the large proteome alteration and the identification of a number of proteins that were only identified in Calr^+/+^ samples and not in Calr^+/−^ ones, and vice versa (Supplementary Materials Figure S4C(I,II)).

### 3.4. Chronic Low Level of Calr Resulted in Calcium Imbalance and Mitochondrial Disorder in Calr^+/−^ Kidney

Each identified protein was assigned to cellular components, functional categories, and biological processes based on the Gene Ontology annotation system using the DAVID Functional Annotation Bioinformatics Microarray Analysis, David Bioinformatics version 6.8, (http://david.abcc.ncifcrf.gov/, accessed on 23 October 2016). Interestingly, a closer look at the proteomics data confirmed the alteration of Ca^2+^ homeostasis in Calr^+/−^ kidney. Several key Ca^2+^ handling proteins from the distal convoluted tubule were significantly altered in their expression in the Calr^+/−^ kidney (Figure 3F–H). The sodium/calcium exchanger 1 (Ncx1) and the plasma membrane Ca^2+^-transporting ATPase 1 (Pmca1) were highly up-regulated in Calr^+/−^ kidneys (Figure 3F,G). Both channels are located in the basolateral plasma membrane of the distal convoluted tubule and are involved in the movement of Ca^2+^ across the membrane. Similarly, the extracellular Ca^2+^-sensing receptor (Casr) was significantly up-regulated in heterozygous mouse kidney (Figure 3F,G). Casr senses changes in the extracellular concentration of calcium ions and plays a key role in maintaining calcium homeostasis. On the other hand, the transient receptor potential cation channel 5 (Trpv5), an epithelial Ca^2+^ channel that constitutes the rate-limiting step of active Ca^2+^ reabsorption in the kidney, was significantly down-regulated (Figure 3G,H). Meanwhile, caveolin-1 (Cav1), a protein involved in endocytosis mediated degradation of Trpv5, was significantly up-regulated in Calr^+/−^, whereas klotho (Kl) and kallirein-1 (Klk1), which increase the apical abundance of Trpv5 in the kidney, were significantly down-regulated (Figure 3F). The increase in the calcium channel expression, which transports the calcium out of the distal convoluted tubule cells, and the decreased expression of proteins that augment the cellular free calcium amount corroborate the fact of the disturbance of cellular calcium homeostasis in the Calr^+/−^ mouse kidney.

**Figure 4:**
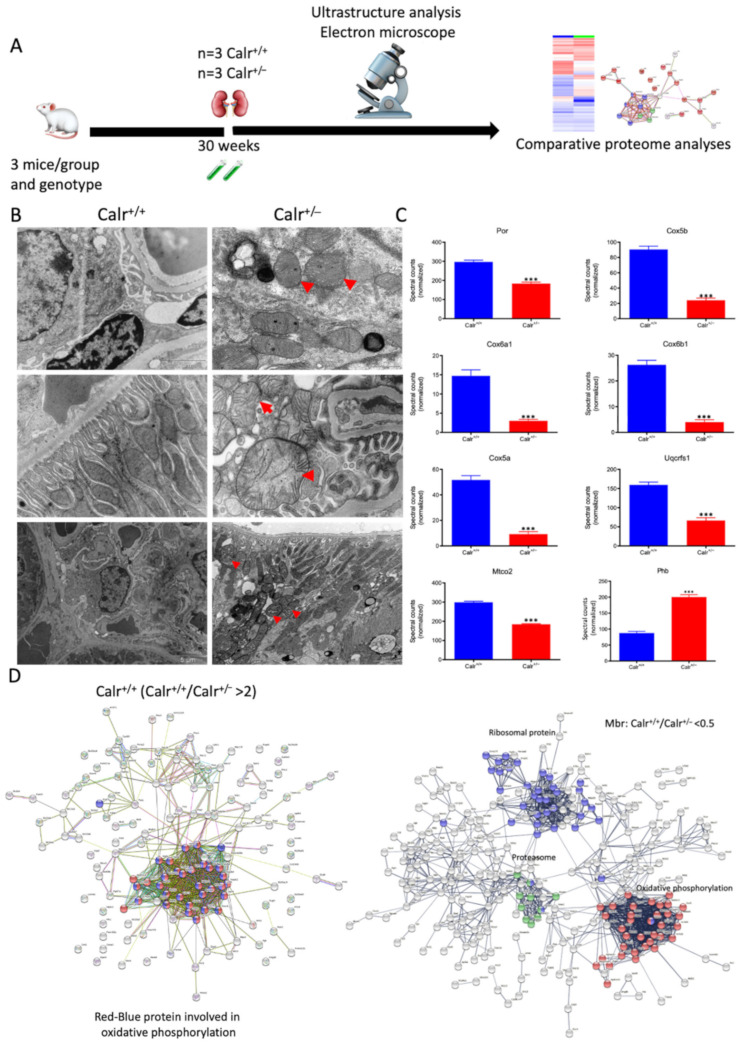

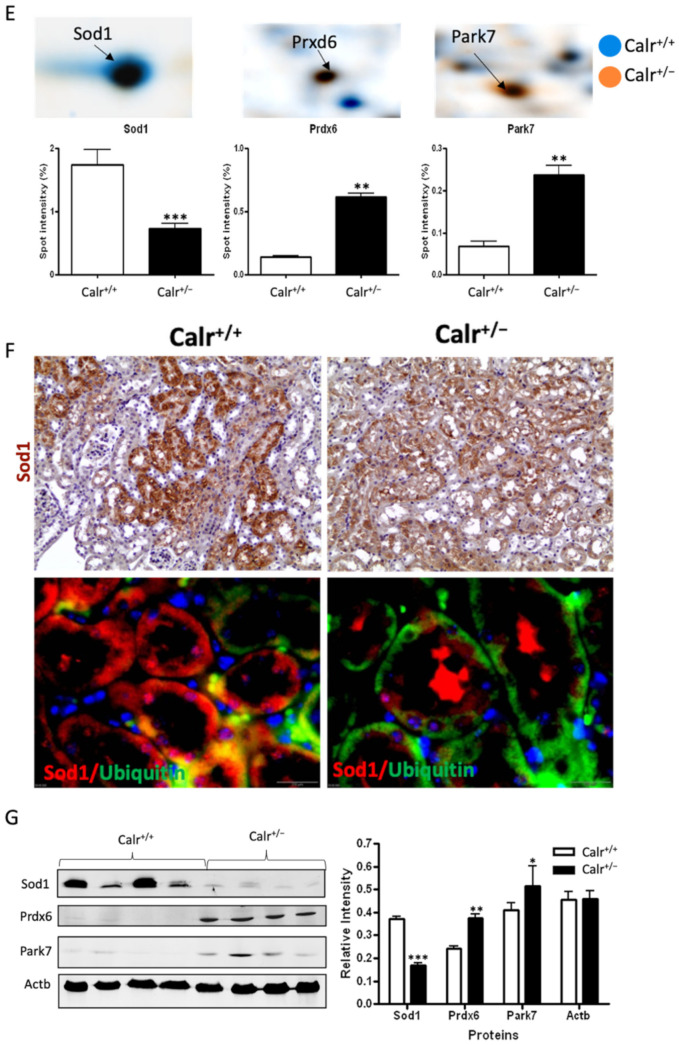
Mitochondrial alteration in Calr^+/−^ mouse kidney. (**A**): Illustration of the experimental design for ultrastructure analysis and comparative proteome investigation. (**B**): Kidney section from Calr^+/+^ and Calr^+/−^ mice were assessed by electron microscopy. Representative electron microscopic images show profound damage in the mitochondria of kidney cells from Calr^+/−^ mice. The micrographs display swelling structures with loss of cristae. (**C**): Proteomics data showing significant down-regulation of proteins involved in oxidative phosphorylation and mitochondrial integrity in Calr^+/−^ mouse kidneys. (**D**): Right: String analysis of the down-regulated proteins in Calr^+/−^ mouse kidneys. String graph shows a strong interaction between the down-regulated proteins. Induction of oxidative stress in Calr^+/−^ mouse kidneys. Left: The up-regulated protein in Calr^+/−^ mouse kidneys are mainly ribosomal proteins, proteins from proteasome, and proteins involved in oxidative phosphorylation. (**E**): 2-DE close-up regions showing regulated protein involved in oxidative stress. (**F**): Immunohistochemical and immunofluorescence staining of Sod1 in Calr^+/−^ and Calr^+/+^ mice. The immunofluorescence staining of Sod1 was coupled with ubiquitin. (**G**): Western blot analysis of oxidative stress-related proteins (Sod1, Prdx6, and Park7) was performed in kidney lysates from Calr^+/−^ and Calr^+/+^ mice. Actb was used as loading control. Bar diagram representing the quantification of the Western blot results shown in G. (*n* = 4, *: *p* < 0.05, **: *p* < 0.01, ***: *p* < 0.001).

High cytosolic Ca^2+^ concentrations induce excessive matrix swelling and lead to the opening of non-selective permeability transition pores, resulting in a wide increase in mitochondrial swelling [28]. To assess the impact of increased cytosolic Ca^2+^ concentration in Calr^+/−^ renal cells on mitochondrial structure, ultrastructural examination of kidney tissues was performed by electron microscopy (Figure 4A). The data showed profound alterations in mitochondrial morphology and number in renal cells of Calr^+/−^ mouse kidneys compared to Calr^+/+^ (Figure 4B). Compared to normal mitochondrial structures of Calr^+/+^ kidney cells, the Calr^+/−^ kidney mitochondria displayed swelling structures with prominent loss of cristae and inner mitochondrial membrane. The latter varied widely in size and shape, from small and rounded to markedly enlarged and swollen with disorganized and fragmented cristae in podocytes. Moreover, tubular cells also showed swelling of several mitochondria with regression of their cristae and an increased number of mitochondria with loss of other cellular structures (Figure 4B).

The 2D gel-based proteomic investigations showed that the largest part of the identified proteins was found to be the mitochondrial (Supplementary Materials Figure S3D). To bring more light to the large set of proteomic data and to get an insight into potential interaction networks involved in kidney injury in Calr^+/−^ mouse kidney, we analyzed the differentially regulated protein in each proteome fraction using String 10.5 (https://string-db.org, accessed on 14 July 2017). In our case, the generated networks showed strong interaction between a large number of regulated proteins. In general, the proteins that were up-regulated in Calr^+/−^ mouse kidney covered three main functional groups: the first group of proteins was involved in mitochondrial structure and the oxidative phosphorylation; the second group in the carboxylic acid metabolism; and the third group in inflammation and innate immune system (Supplementary Materials Figure S4D(I–IV)). The down-regulated proteins in Calr^+/−^ were mainly involved in mitochondrial structural integrity and oxidative phosphorylation (Figure 4C,D). Taken together, the proteomic data provided converging evidence that the chronic low level of calreticulin and the resulting imbalance in calcium homeostasis are accompanied by mitochondrial dysfunction. Moreover, the data suggested an alteration in energy metabolism and increased inflammation and immune response in Calr^+/−^ kidney.

### 3.5. The Mitochondrial Disorder in Calr^+/−^ Mouse Kidney Led on to High-Level Oxidative Stress Resulting in Renal Injury

Excessive ROS production or an inefficient antioxidant system are known as major causes of oxidative stress in the target cells and tissues. The proteomics data showed a down-regulation in the expression of key proteins in oxidative stress. The superoxide dismutase [Cu-Zn] (Sod1) and the glutathione S-transferases (Gstp1, Gstm1, Gsta3, Mgst1, Gsta4, Gsto1, Gstm3, Gstm6) were significantly down-regulated, revealing an increased level of oxidative stress in Calr^+/−^ mouse kidney (Figure 4E, Supplementary Materials Table S2). Simultaneously, the ROS-producing protein from the cytochrome P450 family was significantly down-regulated in Calr^+/−^ mouse kidney (Supplementary Materials Table S2). On the other hand, the oxidative stress proteins peroxiredoxins (Prxd1, Prxd2 Prxd4, Prxd5, and Prxd6) and protein DJ1 (Park7) were significantly up-regulated in Calr^+/−^ mouse kidney, confirming the occurrence of oxidative stress (Figure 4E, Supplementary Materials Table S2). The Prdxs act as a cellular redox control via their ability to eliminate organic hydroperoxides. Their up-regulation in cells and tissues under oxidative stress conditions is the cellular response to rescue oxidative stress and to avoid tissue damage [29].

Western blot analysis from the kidney lysate of each of the four different Calr^+/−^ and Calr^+/+^ mice further confirmed the significant down-regulation of antioxidant Sod1 (Figure 4F,G) and up-regulation of Prdx6 and Park7 proteins in all Calr^+/−^ mouse kidney lysates on an individual basis compared to the Calr^+/+^ mice (Figure 4G). Immunohistochemical staining of kidney tissue slides showed strong expression of Sod1 in the distal tubule in the Calr^+/+^ kidney and weak staining in the rest of the kidney, whereas the staining for Sod1 was even overall in the kidney tissue in the Calr^+/−^ kidney (Figure 4F).

**Figure 5:**
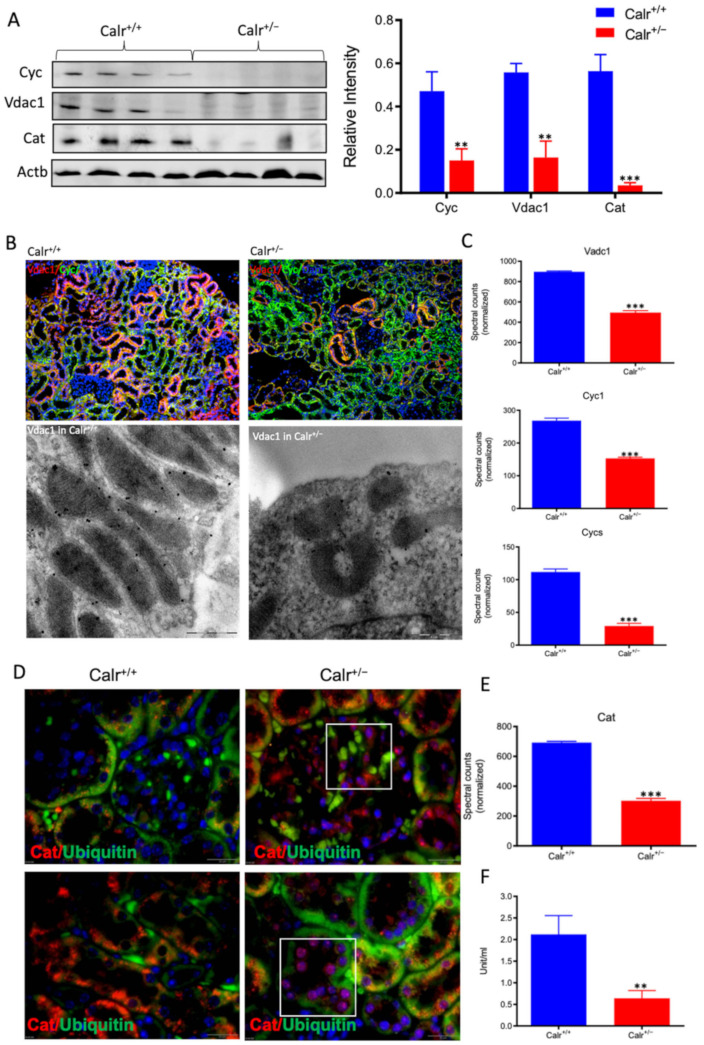
Down-regulation of mitochondrial proteins in Calr^+/−^ mouse kidneys. (**A**): Western blot analysis of mitochondrial proteins: Vdac1, Cyc, and Cat from lysate of enriched mitochondria from Calr^+/+^ and Calr^+/−^ kidney tissues. Quantification of protein expression is shown in bar diagram. (**B**): Immunofluorescence staining of Vdac1 and Cyc shows a clear decrease in the protein expression; micrograph shows the staining of Vdac1 using immunogold. (**C**): Quantification of proteomics data confirming the down-regulation of the investigated mitochondrial proteins. (**D**): Immunofluorescence staining of Cat coupled with ubiquitin shows enhanced expression in the glomerulus and nuclear translocation in the tubule cells of Calr^+/−^ kidneys. (**E**): Quantification of the Cat expression using normalized spectral accounts, the expression of Cat is significantly down-regulated in Calr^+/−^ mouse kidney. (**F**):Quantification of cytochrome c oxidase activity. Intact mitochondria were isolated for the quantification of cytochrome c oxidase activity. Comparison of the respiratory activity between Calr^+/−^ and Calr^+/+^ kidneys revealed about a 50% decrease in mitochondrial activity in Calr^+/−^ kidney cells. ** *p*<0.01, *** *p*<0.001).

Moreover, the Calr^+/−^ kidney appeared to suffer from severe oxidative stress. Investigation of the expression of proteins associated with mitochondria damage revealed significant down-regulation of the expression of the outer membrane channel Vdac1, the mitochondrial oxidative stress marker catalase (Cat), and the cytochrome C (Figure 5A–E). Additionally, cytochrome c oxidase(Cox) activity was determined in isolated mitochondria from kidney tissues, as described in the Material and Methods section. Cox or complex IV of the mitochondrial electron transport chain is the primary site of cellular oxygen consumption and, as such, is of central importance to oxidative phosphorylation and the generation of ATP. The data showed that in addition to impaired expression, a significant decrease in the enzyme activity was measured in the Calr^+/−^ mouse kidney cells exhibiting mitochondrial dysfunction (Figure 5F) and revealing an energy shortage.

### 3.6. Disturbance in Calcium Homeostasis and the Consequent Mitochondrial Disorder Result in Extensive Mitophagy

**Figure 6:**
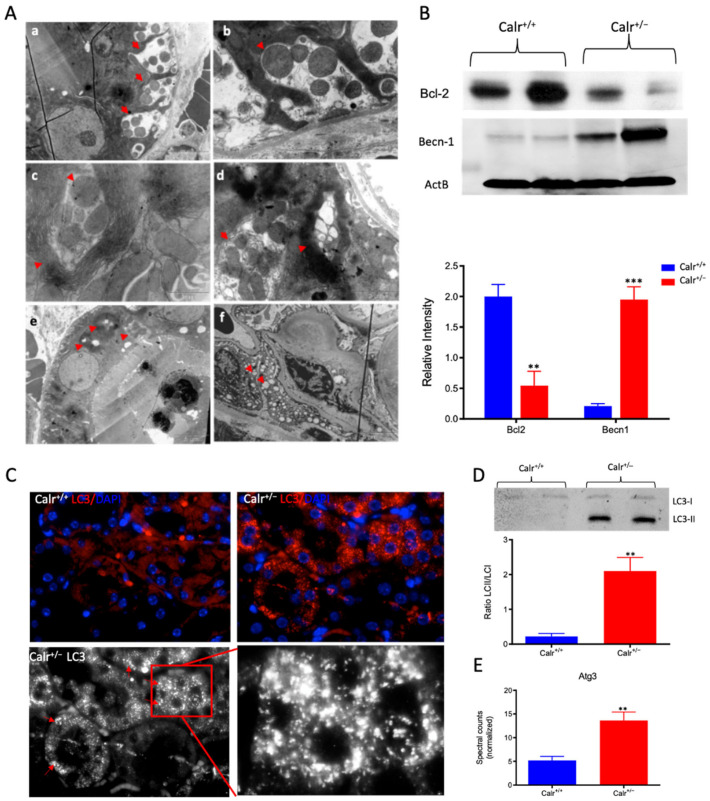
Severe mitochondrial damage and autophagy in Calr^+/−^ mouse kidneys. (**A**): Representative electron micrographs for ultrastructural morphology of mitochondria from Calr^+/−^ mouse kidney—(**a**,**b**): distal convoluted tubule cells swelling mitochondria enclosed in membrane structures, some of the mitochondria are in advanced stages of autophagy; (**c**–**e**): damaged mitochondrial enclosed in multi-membrane structure undergoing autophagy, also shown are advanced stages where the mitochondria are almost completely eliminated; (**f**): a podocyte with damaged vacuolated mitochondria highlighted with red asterisks in Calr^+/−^ mouse kidneys. (**B**): Western blot analysis of protein extract from Calr^+/+^ and Calr^+/−^ kidney tissue showed down-regulation of Bcl-2 and up-regulation of Becn-1, indicating an activation of the autophagy. (**C**): Immunofluorescence staining of LC3 confirmed the initiation and formation of autophagosomes. (**D**): Western blot analysis with antibody against LC3 confirmed the shift toward LC3-II in Calr^+/−^ kidney, as evidenced by the ratio LC3-II/LC3-I calculation. (**E**): Proteomic analysis revealed an up-regulation of the Atg3, an important player in autophagy in Calr^+/−^ kidneys. Results are given as means ± SD of the relative intensity in the case of Western blot analysis, or of the normalized spectral accounts in the case of proteomic data **: *p*<0.01, ***: *p*<0.001).

The chronically increased cytosolic calcium concentration leads to mitochondrial swelling and structural alteration. Interestingly, a closer examination of the ultrastructure of kidney cells showed an extensive mitophagy, especially in the distal tubule (Supplementary Materials Figure S5A). Compared to Calr^+/+^, the mitochondria in Calr^+/−^ renal cells showed an aberrant structure. Moreover, we observed mitophagy at different stages (Figure 6A): aberrant mitochondria enclosed in autophagic vesicles (Figure 6A(a,b,d)), abnormal mitochondria surrounded by multi-membraned autophagic vesicles (Figure 6A(c), Supplementary Materials Figure S5B(a)), mitochondria in progressive degradation and with the presence of myelin-like structures, where the mitochondria are completely eliminated and only empty vesicles are surrounded by the multi-membranes (Figure 6A(d) Supplementary Materials Figure S5B(b)). These provide further evidence of extensive mitophagy in the distal tubule cells. In contrast to the intensive loss through mitophagy, certain tubular cells were densely packed with mitochondria (Supplementary Materials Figure S5B(c)). Moreover, an interesting aspect of mitophagy was observed in the distal convoluted tubule; namely, many aberrant mitochondria were enclosed in large vesicles. These vesicles appear to be in the intercellular area (Figure 6A(d,e), Supplementary Materials Figure S5C(a–f)), as if the cells are eliminating the abnormal mitochondria in the extracellular milieu. In the glomeruli, in addition to the thickening of basement membrane, the cells showed aberrant mitochondria and strong vacuolization (Figure 6A(f), Supplementary Materials Figure S5B(d–f)).

To demonstrate the activation of autophagy in Calr^+/−^, the expression and activation of autophagy markers were investigated. Cell protein extracts were prepared from Calr^+/−^ and Calr^+/+^ kidney tissues and Western blot analyses were performed. Under normal conditions, Bcl-2 inhibits Beclin 1 (Becn1), while in the case of stress (e.g., oxidative stress), Becn1 dissociates from Bcl-2, allowing the activation of autophagy. Our data showed significant down-regulation of Bcl-2 in Calr^+/−^ kidney, while the expression of Becn1 significantly increased, suggesting an activation of autophagy in the Calr^+/−^ mouse kidney (Figure 6B). The microtubule-associated protein 1 light chain 3 (LC3) is an autophagy marker; in particular, the presence of its processed form LC3II is a hallmark for increased autophagy. Staining of LC3 in kidney tissue from Calr^+/+^ and Calr^+/−^ mice confirmed the presence of many autophagosomes in Calr^+/−^ tubule cells, which appear as LC3 positive puncta (Figure 6C). Moreover, Western blot analysis confirmed a significant increase in LC3II in Calr^+/−^(Figure 6D) and the proteomic data demonstrated an increased expression of Atg3 (Figure 6E), the protein responsible for LC3 processing to LC3II.

### 3.7. The Excessive Mitochondrial Damage in the Calr^+/−^ Mouse Kidneys Results in Impaired Oxidative Energy Metabolism and Activation of Glycolysis

**Figure 7:**
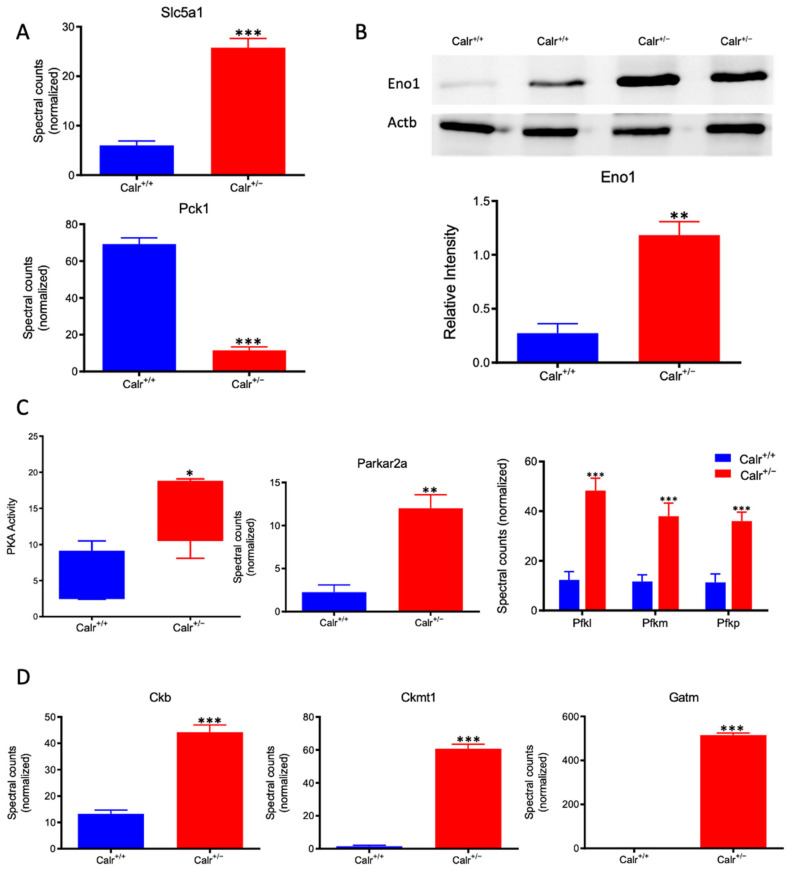
Activation of PKA and glycolysis in Calr^+/−^ kidney. The intensive mitochondrial damage results in an energy shortage. To overcome the energy crisis, the kidney cells in Calr^+/−^ mice activate glycolysis. (**A**): Up-regulation of Slc5a1 a sodium/glucose cotransporter 1, which actively transports glucose into the cell, and PcK1 is also up-regulated to actively augment the glucose synthesis from lactate. (**B**): Enolase 1 is also up-regulated to favor the energy production from glucose. (**C**): PKA is significantly up-regulated and activated to accelerate glycolysis and to promote energy production, and the important glycolysis kinase PFK is significantly up-regulated in Calr^+/−^ mouse kidneys. (**D**): Parallel to the activation of glycolysis, the enzymes involved in production of the alternative energy from phosphocreatine are also up-regulated in Calr^+/−^ mouse kidney. data (*: *p* < 0.05) **: *p* < 0.01, ***: *p* < 0.001).

Calr^+/−^ kidney showed a strong alteration in mitochondrial structure and mitochondrial protein expression and suffered from a low level of oxidative energy. To demonstrate whether the renal cells in the Calr^+/−^ kidney activate an alternative pathway to cope with the energy shortage problem, the activation of glycolysis was investigated. Proteomic data revealed a significant up-regulation of key players in glucose metabolism; the sodium/glucose cotransporter 1 (Slc5ca1) and Phosphoenolpyruvate carboxykinase (Pck1), which catalyze the rate-limiting step in the gluconeogenesis and alpha-enolase (Eno1), were significantly up-regulated in Calr^+/−^ kidney (Figure 7A,B). Moreover, the activity assay of protein kinase A (Parkar2a) demonstrated significant activation of the kinase in the Calr^+/−^ kidney, and the proteomic data confirmed an increase in the abundance of the protein supporting the activation of glycolysis to compensate for the low level of energy and the extensive mitochondrial loss (Figure 7C). Interestingly, the mitochondrial creatine kinase (Ckmt1), the creatin kinase B (Ckb), and the Glycine amidinotransferase (Gatm), the key proteins of the metabolism of phosphocreatine, which is the temporal and spatial energy buffer to maintain cellular energy homeostasis, were highly expressed in Calr^+/−^ kidney for reinforcing the compensatory energy rescue attempts (Figure 7D).

### 3.8. The Increased Cytosolic Calcium Concentration Involves Three Cytosolic Calcium Sensors and Activates the NF-κB -Pathway

**Figure 8:**
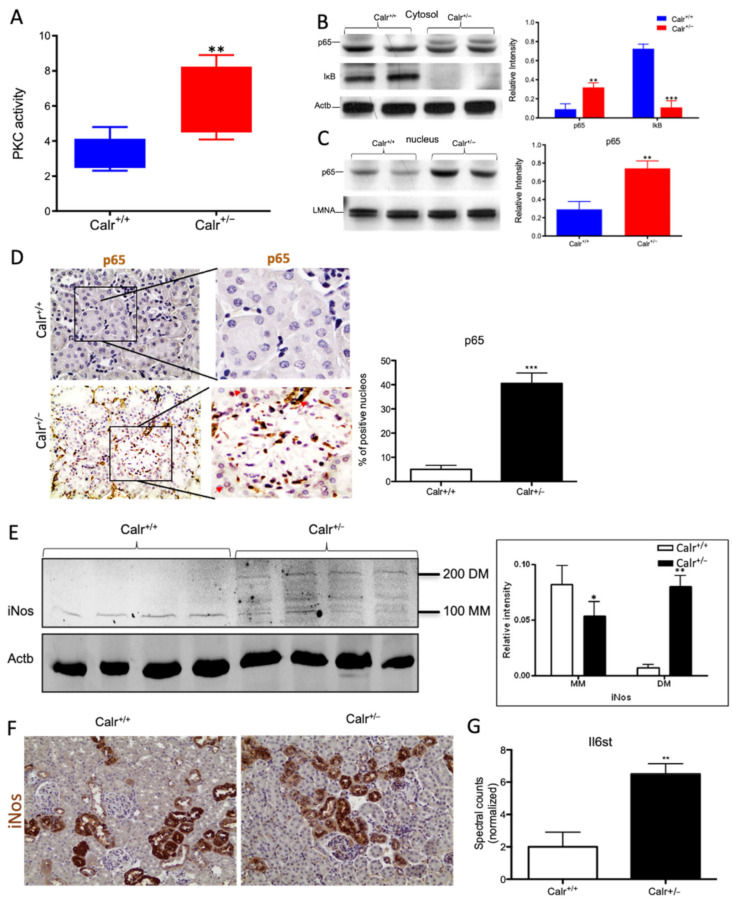
Chronic increased cytosolic calcium level results in activation of PKC and NF-κB pathway in Calr^+/−^ kidney. (**A**): Activity assay demonstrated significant activation of PKC in Calr^+/−^ mouse kidney because of alteration in cytosolic calcium concentration. (**B**): Western blot analysis of protein extracts from Calr^+/+^ and Calr^+/−^ kidneys showed an activation of NF-κB pathway as evidenced by up-regulation of p65 in heterozygous kidney and down-regulation of the pathway inhibitor IkB. (**C**,**D**): The nuclear translocation of p65 confirmed the activation of NF-κB pathway, the nuclear protein Lamin A/C was used as control. (**E**): Western blot analysis of iNos was performed for kidney lysates of Calr^+/−^ and Calr^+/+^ mice. Actb was used as loading control. Bar diagram representing the quantification of the MM and DM of iNos. Western blot results are shown in (**B**) (*n* = 4 *, *p* < 0.05, **: *p* < 0.01, ***: *p* < 0.001). MM: monomer, DM: dimer. (**F**): Immunohistochemical staining of iNos shows no significant change in expression pattern of protein in Calr^+/−^ compared to Calr^+/+^. (**G**): Proteomic data showed an up-regulation of IL6st the interleukin-6 receptor, revealing an increased inflammation upon NF-κB pathway activation in Calr^+/−^ mouse kidney.

The repressed expression of Regucalcin (Rgn) (Supplementary Materials Figure S5D), a suppressor of Ca^2+^ signal transduction, in Calr^+/−^ kidney revealed an aberrant activation of the calcium signaling. Rgn is known to have an inhibitory effect on Ca^2+^/calmodulin-dependent protein kinase (CamK2) and on protein kinase C (PKC) [30,31]. Moreover, Rgn suppresses the nitric oxide synthase to activate Sod1 [30]. The Calr^+/−^ kidney expresses higher levels of calmodulin-1 (Calm1) and calcium/calmodulin protein kinase (Camk2) when compared to Calr^+/+^, indicating the activation of this pathway. Moreover, an increased expression of the inositol 1,4,5-trisphosphate receptor type 1 (Itpr1) and the inositol 1,4,5-trisphosphate receptor type 2 support the abnormal activation of calcium signaling in the Calr^+/−^ kidney (Figure 8A). One of the most important effectors of alteration in cytosolic Ca^2+^ concentration is PKC. The activity assay of protein kinase C demonstrates a significant activation of the kinase in Calr^+/−^ kidney (Figure 8A) and the up-regulation of 1-phosphatidylinositol 4,5-bisphosphate phosphodiesterase (Plc), the phospholipase catalyzing the production of diacylglycerol (DAG), the activator of PKC, substantiates the significant activation of protein kinase C in the Calr^+/−^ kidney.

Our data demonstrated that the cellular sensors of cytosolic Ca^2+^ levels, calmodulin, PKC, Itrp1, and the Itpr2, are simultaneously activated in Calr^+/−^ kidney. These calcium sensors are involved in connecting the Ca^2+^ second messenger to the NF-κB pathway [32]. To demonstrate whether the NF-κB signaling is involved in Calr^+/−^ kidney pathology, we investigated the key proteins of the pathway in Calr kidney. Apparently, the activation of the calcium sensors resulted in the up-regulation of the p65 subunit of NF-κB in the Calr^+/−^ kidney, while the inhibitor of NF-κB (IκB) was significantly down-regulated (Figure 8B). Western blot analysis of the nuclear fractions and the immunohistochemical staining of kidney tissue confirmed the nuclear translocation of p65 and the activation of the NF-κB-pathway in the Calr^+/−^ kidney (Figure 8C,D). As a result of the activation of the NF-κB pathway, induced nitric oxide synthase (iNos) was activated, as evidenced by its dimerization (Figure 8E,F) and the interleukin-6 receptor (Il6st) was up-regulated (Figure 8G). Both are key players in NF-κB triggered tissue inflammation.

### 3.9. The Activated NF-κB Pathway Results in Inflammation and Injury in Calr^+/−^ Kidney

**Figure 9:**
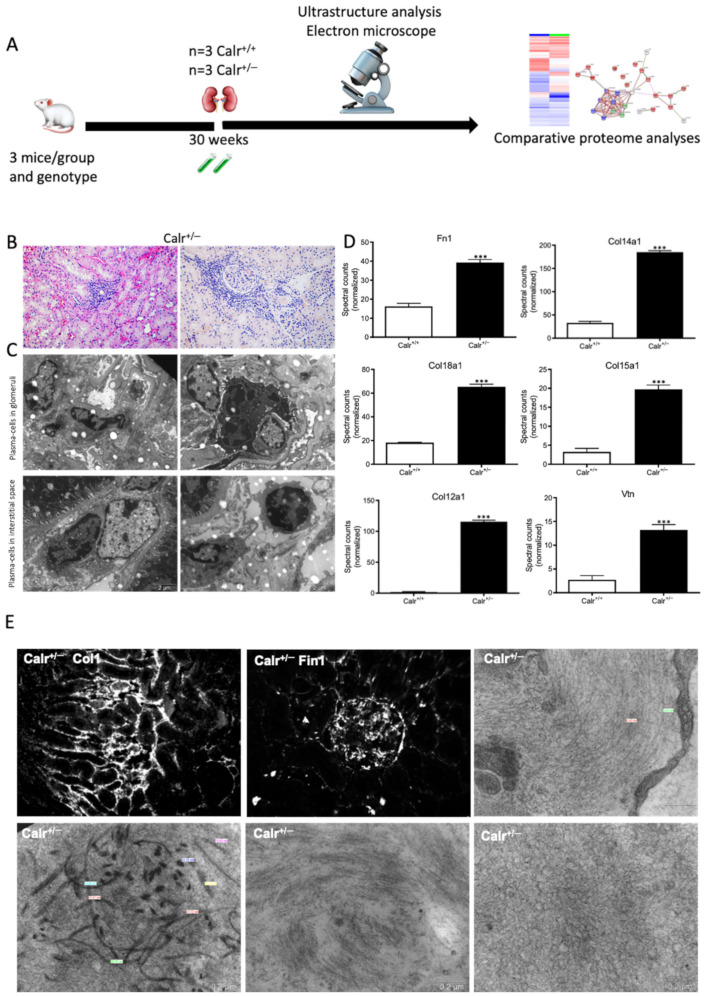
Blood cell infiltration and inflammation of kidney tissue in Calr^+/−^ mice. (**A**): Illustration of the experimental design showing samples processing for structural analysis and comparative proteome investigation. (**B**): Histochemical staining showing strong inflammation in the Calr^+/−^ kidney tissue. **C**: Ultrastructural analysis with electron microscope showed strong infiltration of blood cells in Calr^+/−^ kidney tissue. (**D**,**E**): proteomic data showed significant up-regulation of ECM proteins and the immunofluorescence staining and electron microscopy analysis demonstrated a strong deposition of EMC in the interstitial area of the kidney tissue in Calr^+/−^ mice. ***: *p* < 0.001.

**Figure 10:**
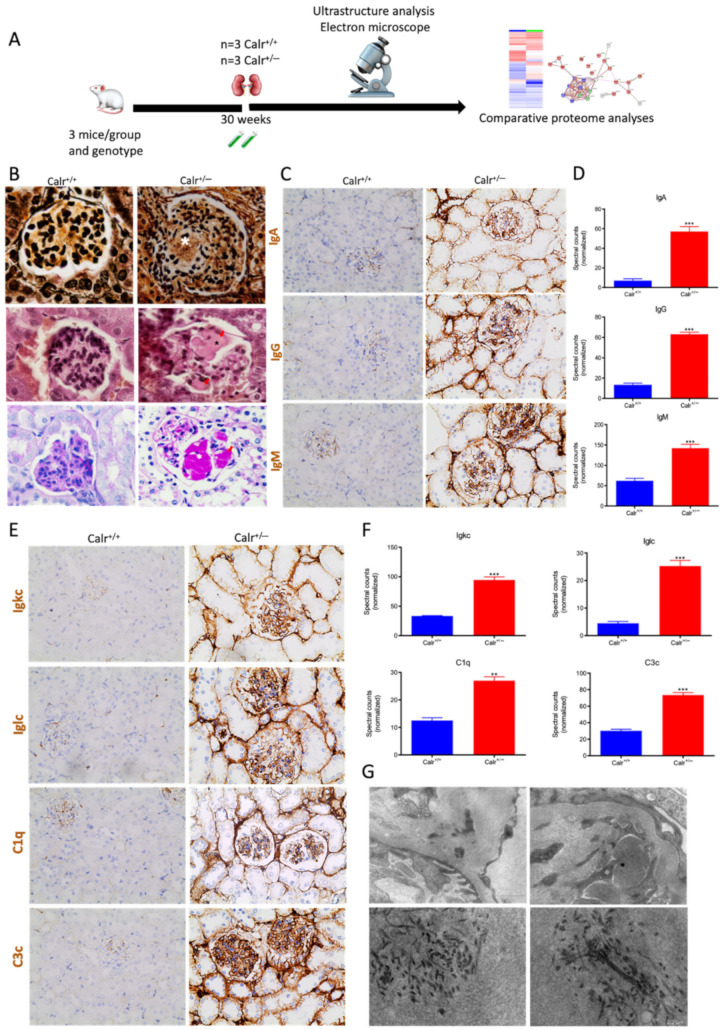
Calr^+/−^ kidney showed an anti-GBM disease. (**A**): Illustration of the experimental design showing samples processing for structural analysis and comparative proteome investigation. (**B**): Histochemical staining shows the typical strong linear ribbon-like appearance, revealing an anti-GBM disease. (**C**): Immunohistochemical staining showing positive staining of IgA, IgG, IgM, in Calr^+/−^ kidney tissue. (**D**): Proteomic analysis confirmed the up-regulation of the three investigated proteins. (**E**): The staining pattern was similar for Igkc, Iglc, C1q, and C3c, with positive staining in Calr^+/−^ kidneys and almost no stain detected in Calr^+/+^ kidneys. (**F**): Proteomics data confirmed the staining results and showed an up-regulation of the complement factor proteins and the light chains. (**G**): Electron microscopic photograph of the Calr^+/−^ renal tissue, showing the electron-dense deposits in mesangial areas. **: *p* < 0.01, ***: *p* < 0.001.

The activation of the NF-κB pathway triggered an invasion of inflammatory cells. Both interstitial and glomerular inflammatory infiltrate were detected (Figure 9A–C). Moreover, the key proteins involved in the transcellular plasma cell infiltration were found to be up-regulated in Calr^+/−^ kidney (Supplementary Materials Figure S5E,F). The extensive inflammation was accompanied by an excessive accumulation of extracellular matrix and thickening of the basement membrane in the Calr^+/−^ kidney (Figure 9D,E). Electron microscopic photograph and histological staining showed strong deposits in mesangial areas and filamentous structures (Figure 9E and Figure 10A,B).

Immunohistochemical staining of Calr^+/−^ and Calr^+/+^ kidney tissues showed positive staining of IgA, IgG, IgM, Igkc, Iglc, C1q, and C3c. IgA and IgG showed a strong reaction along the glomerular basement membrane, resulting in a strong linear ribbon-like appearance, and an up-regulation in the expression was also observed (Figure 10C,D). IgM, Igkc, and Iglc showed staining along the capillary wall but also in the mesangial area (Figure 10E,F), whereas C1q and C3c showed a diffuse mesangiocapillary pattern (Figure 10E). Ultrastructural analysis with electron microscopic showed a photograph with intense electron-dense deposits in mesangial areas accompanying the filamentous structures revealing a deposition of immunocomplexes in the basement membrane and mesangial space (Figure 10G).

## 4. Discussion

Calr is known as the major Ca^2+^-binding, -storage, and -buffering protein in ER [33]. Staining of mouse kidney tissue from wild type showed that Calr is highly expressed in distal convoluted tubules and presents a weak expression in the proximal tubule, whereas in the glomerular area, Calr could not be detected. Calr^−/−^ is lethal, and embryos die because of impairment in heart development [17,34]. In contrast, Calr^+/−^ mice present normal phenotype but express a lower level of Calr; in kidney tissue, the expression of Calr was about 50% lower than in Calr^+/+^. The chronic low level of Calr resulted in a pathological manifestation in the kidney in more than 50% of the investigated cases. The pathological renal alteration in Calr^+/−^ mice can be attributed to one of the main functions of Calr: either to improper protein folding, leading to ER stress, or to a disturbance in Ca^2+^ storage, resulting in increased cytosolic free Ca^2+^ and cytotoxicity. However, investigation of ER proteins and UPR pathway showed no alteration in the expression of the ER stress markers Grp78 and Erp57 or UPR markers p-eIF-2A and Chop, which ruled out the role of ER stress as a causal factor for renal damage in Calr^+/−^ kidney. Calcium investigations demonstrated a disturbance in Ca^2+^ homeostasis. Calr^+/−^ mice suffer from an alteration in ER Ca^2+^ calcium storage resulting in the accumulation of toxic Ca^2+^ levels in the cytosol. Interestingly, the proteins involved in calcium handling, sensing, and reabsorption were subjected to significant expression alteration in Calr^+/−^ mouse kidneys; these showed a significant up-regulation of a group of EF-hand cytosolic Ca^2+^-binding proteins. These proteins, which contain EF-hand motifs, are Ca^2+^ sensors and are mainly involved in Ca^2+^ buffering in the cytosol [35], strengthening the Ca^2+^ handling problem in Calr^+/−^ mouse kidney.

Ca^2+^ reabsorption in the kidney can be paracellular or transcellular. The latter takes place in distal tubule (DT) cells and is regulated by PTH and VD3 [36,37]. Ncx1 and Trpv5 play an important role in this process [38,39]. Both proteins are highly expressed in DT. Their expression was regulated by PTH and VD3 and was significantly altered in the Calr^+/−^ kidney. In transcellular Ca^2+^ reabsorption, the calcium-binding protein Calb1 plays a key role in the transit of Ca^2+^ through the epithelial cells from the apical side to the basolateral side without significant modification in the intracellular Ca^2+^ [36]. In Calr^+/−^ kidneys, Calb1 was significantly up-regulated. Similar to Trpv5, Calb1, and Ncx1, we demonstrated that Calr expression underlies the control of the calcium-regulating hormones PTH and VD3 in distal tubule cells. Taken together, our data suggest that Calr is an important piece in the puzzle of transcellular calcium handling in the kidney distal tubule and that the chronic low level of Calr results in increased cytosolic calcium and kidney damage.

Increases in cytosolic free Ca^2+^ represent a ubiquitous signaling mechanism that controls a variety of cellular processes, including proliferation, metabolism, and gene transcription. Yet, abnormal and chronic increases in intracellular Ca^2+^ are cytotoxic and cause pathological manifestation. The mitochondrion plays an important role in the rapid sequestering of Ca^2+^ during the development of the Ca^2+^ signal and then slowly releases it back during the recovery phase. This uptake of Ca^2+^ by the mitochondrion is important in shaping both the amplitude and the spatio-temporal patterns of the Ca^2+^ signals [40]. Moreover, modifications in cytosolic Ca^2+^ concentration are known to directly correlate with changes in mitochondrial energy metabolism and ATP production through interaction with oxidative phosphorylation and electron transport chain enzymes [41,42]. Alteration in cytosolic Ca^2+^ concentration may result in mitochondrial dysfunction, oxidative stress, and cause pathological manifestation [41,42]. In Calr^+/−^ mouse kidney, especially in the distal convoluted tubule, the alteration of ER Ca^2+^ storage and the continued uptake of calcium from tubule lumen resulted in an increase in the cytosolic free Ca^2+^ concentration, leading to an uncontrolled Ca^2+^ uptake in the mitochondria and to mitochondrial swelling. This induced mitochondrial damage and oxidative stress, resulting in the impairment of oxidative phosphorylation and ATP production. Decreased cytochrome c oxidase activity further confirmed the loss of mitochondrial function accompanied by an energy crisis in Calr^+/−^ mouse kidneys. Previous studies have shown that the ability of the cells to utilize different metabolic pathways to support energy production is critical for survival under stress conditions; if compromised, this activates the programmed cell death and their degradation by autophagy, a phenomenon whereby cells can digest themselves [43]. In Calr^+/−^ mouse kidneys, the alteration in Ca^2+^ homeostasis and increased mitochondrial aberrations led to abnormal activation of autophagy and the significant pathological alteration of kidney tissues. Particularly striking was the presence of autophagosomes at different stages and with a large number of mitochondria. Additionally interesting were the number and shapes of these autophagosomes in tubular cells, highlighting the immense mitochondrial disorder the cells suffer from. To compensate for the energy shortage resulting from the loss of mitochondria, the Calr^+/−^ mouse kidney cells activated the PKA pathway and glycolysis as alternative metabolic pathways to counteract the energy shortage.

Besides mitochondrial disease, Calr^+/−^ kidney cells suffered from abnormal activation of the calcium signaling, resulting in impairment of kidney cell function. Indeed, Calr^+/−^ kidney cells activated PKC and the NF-κB pathways, which in turn activated the inflammatory cascades, resulting in blood cell infiltration, deposition of immune complexes in the glomerular and tubular basement, and massive kidney tissue injury.

## 5. Conclusions

The kidney, especially the distal convoluted tubule, plays a key role in the fine-tuning of Ca^2+^ homeostasis. Ca^2+^ reabsorption in this tubule part is subjected to PTH control. We demonstrated that Calr, through its calcium binding and storing capacities, plays a key role in the fine-tuning of Ca^2+^ homeostasis. Chronic alteration in the level and function of Calr results in the disorder of Ca^2+^ homeostasis, impairment of the entirety of pathways involved in oxidative stress, alteration of mitochondrial structure and function, shortage of energy metabolism, and is linked to the pathology of renal injury.

## Data Availability

Not applicable.

## Supplementary Materials

The following supporting information can be downloaded at: https://www.mdpi.com/article/10.3390/cells11081329/s1, Table S1: Oligonucleotide sequences for canis lupus Calr single guide RNA; Table S2: Glutathione S-transferases, peroxiredoxins, and Cytochrome P450 that were significantly regulated in Calr^+/−^ mice kidney; Figure S1: Morphometric and histological analysis of kidney from Calr^+/+^ and Calr^+/−^ mice. **A**: Illustration of the experimental design for morphological and immunohistological analysis. **B**: Gross morphology of kidneys from 40-week-old Calr^+/+^ (left) and Calr^+/−^ (right) mice showing an enlargement of Calr^+/−^ kidney compared to Calr^+/+^. Bar diagram represents the significant increase in Calr^+/−^ kidney weight at 40 wk of age. **C**: Longitudinal sections of kidneys showing progressive internal impairments indicated with arrows (yellow) in Calr^+/−^ mice. Genotypes are indicated at the top. wk: weeks. **D**: Paraffin-embedded kidney sections (3 µM) were stained with PAS to compare the kidney structures of Calr^+/+^ and Calr^+/−^ at 30 wk of age. The PAS staining revealed progressive tubular atrophy (red arrows) in Calr^+/−^ kidney, the distal convoluted tubule is especially affected. Glomerular injury is also advanced, showing an expansion of the mesangium with accumulation of PAS-positive material in Calr^+/−^ mice (magnification ×20). **E**: Tubulointerstitial area stained with PAS staining revealed progressive tubulointerstitial damage in Calr^+/−^ mice compared to Calr^+/+^ mice (magnification ×20). **F**: Representative electron micrographs for ultrastructural morphology of kidney section from 15- and 40-week-old Calr^+/−^ mice. Images show damaged structures in kidney from 40-week-old Calr^+/−^ mice compared with younger animals; Figure S2: Increased calcium concentration and level of calcium handling hormone in Calr^+/−^ serum. **A**: Illustration of the experimental design for calcium investigation in serum urine. **B**: (**I**), Calcium concentration was estimated in serum of Calr^+/+^ and Calr^+/−^ mice. The data showed a significant increase in calcium concentration in serum of Calr^+/−^ mice, the alteration is age dependent. The calcium level in serum of old Calr^+/−^ mice was significantly higher than in serum of Calr^+/+^ and young Calr+/− mice. (**II**), Urine calcium concentration in Calr^+/+^ and Calr^+/−^; no significant difference in calcium excretion could be observed between Calr^+/+^ and Calr^+/−^. **C**: chloride (**I**) and sodium (**II**) were estimated in urine samples (*n* = 30 each) using standard routine laboratory methods. No significant difference in chloride and sodium excretion could be measured between Calr^+/+^ and Calr^+/−^ samples. **D**: PTH (**I**) and VD3 (**II**) estimations were carried out in serum of Calr^+/+^ and Calr^+/−^ mice using ELISA. **E**: Staining of free calcium in primary cell isolated from Calr^+/−^ and Calr^+/+^ kidney using cell permeant Fura-2. Primary cells from Calr^+/−^ kidney showed increased free Ca^2+^ level than the cells from WT kidney. **F**: Intact kidney slices were prepared according to Crawford et al. [27], the perfused kidney slices were loaded with Fura-2 at room temperature for 45 min. The Calr+/− tissue showed an increased free calcium level compared to Calr^+/+^. **G**: Western blot analysis of MDCK protein extract with antibody against Calr showing significant down-regulation in MDCK-CalrKo compared to control. **H**: qPCR confirmed the significant down-regulation of Calr in MDCK-CalrKo cells. **K**: Treatment of MDCK-CalrKo and MDCK-controls with ATP and the calcium inophor A23187 confirmed that the Calr deficiency resulted in impairment of ER calcium storage and the ability to release calcium to cytosol upon stimulation; Figure S3: 2-DE based comparative proteome analysis of the kidney from Calr^+/+^ and Calr^+/−^ and Gene Ontology (GO) classification of differentially regulated proteins. **A**: Illustration of the experimental procedure for the kidney proteome using 2-DE and mass spectrometry. **B**: Overlapping 2-DE map of Calr^+/+^ and Calr^+/−^ kidneys proteomes. Blue spots indicate higher expression in Calr^+/+^ mouse kidney samples compared to Calr^+/−^ mice samples. Orange spots indicate an up-regulation of the proteins in Calr^+/−^. Proteins expressed in both phenotypes are overlapping spots and are shown in black. C: Magnified images of regions of interest showing differentially regulated proteins in the Calr^+/−^ mouse kidney. The protein expression quantification for selected proteins is given in form of bar diagrams. Results are given as the means ± SD of the percentage volume of spot from at least three independent experiments (*p* < 0.05). Figure S4: Deep analysis of Calr^+/+^ and Calr^+/−^ kidney proteomes. **A**: The experimental procedure for the deep kidney proteome analysis. Fractionation of the kidney tissue proteome in sub-proteomes resulted in 4 fractions: membrane, mitochondrial, cytosolic, and nuclear. The fraction proteomes were analyzed combining 1DE and mass spectrometry. **B**: **I**–**IV**: Volcano plot analysis of the identified and quantified proteins allowed the easy identification of proteins differentially expressed between Calr^+/+^ and Calr^+/−^ kidney. **C**: **I**, **II**: Pie diagram presentation of the protein identified in the different fractions. The pie diagram allows for identifying proteins expressed only in one phenotype or the overlapping proteins that were identified in both phenotypes. **D**: **I**–**IV**: Protein interaction networks between the proteins found to be up-regulated in Calr^+/−^ mouse kidney were generated using the protein networks software String (https://string-db.org). Figure S5: Ultrastructural analysis of kidney tissue demonstrating intensive autophagy in Calr^+/−^ kidney **A**: Illustration of the experimental design for ultrastructure analysis and comparative proteome investigation. **B**: Representative electron micrographs for ultrastructural morphology of mitochondria from Calr^+/−^ kidney. Tubular cells with autophagosomes containing mitochondria (**a**), or mitochondria in advanced stage of autophagy, a number of mitochondria are enclosed in a membranous network in tubular cell, a progressive autophagosome with completely damaged mitochondria (**b**), robust number of mitochondria in certain tubular cells (**c**). Podocyte with damaged vacuolated mitochondria highlighted with red asterisks (**d**), higher magnification image of a podocyte illustrating mitochondrial swelling and damage with disordered cristae indicated with red arrows (**e**), mitochondrial swelling in a tubular cell indicated with an arrow (**f**). **C**: Autophagosome with mitochondria (**a**–**c**), accumulation of swelling mitochondria in large membrane structure (**d**–**f**). **D**: Analysis of proteomic data showed a down-regulation of regucalcin, an inhibitor of Camk2 and PKC, while Calm-1 and Camk2 were up-regulated. E, F: Quantification of proteomics data demonstrating an up-regulation in the expression of the key proteins involved in the transcellular plasma cell infiltration in Calr^+/−^ kidney.
