# Supplementary material for: Calreticulin Shortage Results in Disturbance of Calcium Storage, Mitochondrial Disease, and Kidney Injury
Source: Cells. 2022 Apr 13;11(8):1329. doi: 10.3390/cells11081329 (PMC9025518; doi:10.3390/cells11081329)
Supplement: Supplementary file 1 [file cells-11-01329-s001.zip › cells-1617540-supplementary.pdf]

Type of the Paper: Article

Title: Calreticulin shortage results in disturbance of calcium storage, mitochondrial disease, and kidney injury

Asima Tayyeb[1#], Gry H. Dihazi[2#], Björn Tampe[3], Michael Zeisberg[3], Desiree Tampe[3], Samy Hakroush[4], Charlotte Bührig[3], Jenny Frese[5], Nazli Serin[3,6], Marwa Eltoweissy[7], Gerhard A. Müller[3], Hassan Dihazi[3,8*]

[1] School of Biological Sciences, University of the Punjab Lahore, Pakistan; asima.sbs@pu.edu.pkm
[2] Institute for Clinical Chemistry/UMG-Laboratories, University Medical Centre Göttingen, Robert-Koch-Strasse 40, 37075 Göttingen, Germany; gryhelene.dihazi@med.uni-goettingen.de
[3] Clinic for Nephrology and Rheumatology, University Medical Centre Göttingen, Robert-Koch-Strasse 40, 37075 Göttingen, Germany; bjoern.tampe@med.uni-goettingen.de; michael.zeisberg@med.uni-goettingen.de; desiree.tampe@med.uni-goettingen.de; gmueller@med.uni-goettingen.de; charlotte.buehrig@gmx.de; nazli.serin@med.uni-goettingen.de; dihazi@med.uni-goettingen.de
[4] Department of Pathology, University Medical Centre Göttingen, Robert-Koch-Strasse 40, D-37075 Göttingen, Germany; samy.hakroush@googlemail.com
[5] Department of Occupational Medicine and Health Safety, Deutsche Post AG Kölnische Strasse 81, 34117 Kassel Germany; jenny.frese@med.uni-goettingen.de
[6] University of Medical Center Göttingen; Department of Hematology and Oncology, University Medical Centre Göttingen, Robert-Koch-Strasse 40, Germany; nazli.serin@med.uni-goettingen.de
[7] Department of Zoology, Faculty of Science, Alexandria University, Alexandria, Egypt; marwaeltoweissy@alexu.edu.eg
[8] Centre for Biostructural Imaging of Neurodegeneration (BIN), University Medical Center Göttingen, 37075 Göttingen, Germany; dihazi@med.uni-goettingen.de
* Correspondence: dihazi@med.uni-goettingen.de; Tel.: +49 551 3960350
# This Authors contributed equally to this work

1. Supplemental Material and Methods

1.1. Design of sgRNA oligonucleotides for CRISPR/cas9 in MDCK cells

The sgRNA sequences for Calreticulin (ENSCAFT00000027074.3, XM8622117) from Canis Lupus were designed using the online tool Blueheronbio (Origene, Herford, Germany). The sgRNA sequence 5′ GTAGATGGCGGGTTCGGCAG 3′ were inserted into the pLenti-Cas-Guide plasmid (Origene, Herford, Germany) with BamHI and BsmBI restriction enzymes to generate pLenti-Cas-Calr construct later confirmed by Sanger sequencing. The plasmid encoding Plenti- Cas-Guide were purchased from

Addgene (Addgene plasmids). The primer sequences used for PCR cloning are listed in Supplemental Table 2.

Supplemental Table S1: Oligonucleotide sequences for canis lupus Calr single guide RNA

**sgRNA_dgCalrF1:**	GATCGTAGATGGCGGGTTCGGCAG
**sgRNA_dgCalrR1:**	AAAACTGCCGAACCCGCCATCTAC
**sgRNA_dgCalrF2:**	GATCGCTGGTCATTGTAGAACTTGC
**sgRNA_dgCalrR2:**	AAAAGCAAGTTCTACAATGACCAGC

1.2. Cell Culture and Plasmid transfection of the MDCK cells

Madin-Darby canine kidney (MDCK) cells were obtained from American Type Culture Collection (ATCC) and propagated in Dulbecco's Modified Eagle's Medium (DMEM), 10% fetal bovine serum (FBS), 1% Penicillin/Streptomycin, 1% L-Glutamine at 37°C in a 5% CO2 atmosphere. Cells are cultured in T25 flasks and splitted twice in a week.

In order to knockout calreticulin in MDCK cells, Plenti-Cas-Calr transfection were performed. One day before the transfection 200.000 cells/ml were seeded in 6 well plates with MEM medium. Before the transfection medium was changed to FCS-free medium/Opti-MEM medium. Lipofectamine 2000 (Invitrogen, USA) was used for transfecting the cells. Lipofectamine plasmid DNA mix prepared with Opti-MEM based on the instructor's protocol. For 6 well plate transfection, 4µg plasmid DNA was added to 250 µl Opti-MEM medium separately and incubated 10 min at room

temperature. Subsequently the DNA mixture was added to Lipofectamine 2000 mixture and incubated for 30 min before been added to the cells.

1.3. Protein extraction, precipitation and estimation

Kidneys were homogenized in buffer containing Tris-HCl 50 mmol/L (pH 7.4), 1% Triton X-100, 100 mmol/L NaCl and protease inhibitors. After incubation for 30 min at 4°C, kidney tissue homogenates were centrifuged two times at 14,000 rpm for 30 min, and the supernatant was collected. To reduce the salt contamination and to enrich the proteins, protein precipitation was performed. Whole tissue homogenate was precipitated by methanol-chloroform as previously described by (1). The precipitation eliminates lipids, nucleotides, and salts, which improves the resolution of 2D gel analysis (2). Protein concentration was measured according to Bradford assay (3), using bovine serum albumin as a standard.

1.4. 2-D gel electrophoresis (2-DE)

2-D gel electrophoresis (2-DE) analysis of $Calr^{+/-}$ kidneys compared to WT kidneys was performed according to (4). Briefly, a total protein concentration of 150 μg in rehydration buffer (8 M urea, 1% CHAPS, 1% DTT, 0.2% ampholytes, and a trace of bromphenol blue) was loaded on 11-cm IPG strips pH 5-8 from Bio-Rad (Hercules, CA) using passive rehydration at 20 °C. Isoelectric focusing was performed using the Protean IEF cell (Bio-Rad) for 50,000 Vh. After equilibration, IPG strips were loaded on 12% BisTris Criterion precast gels (Protean Xi, Bio-Rad) and run at 200 V for second dimension separation of proteins.

For image analysis, 2D gels were fixed in a solution containing 50% methanol and 12% acetic acid for 2 h and stained with Flamingo fluorescent gel stain (Bio-Rad, Hercules, CA, USA) for minimum 5 h. After staining, gels were scanned at 50 μm resolution on a Fuji FLA5100 scanner. The digitalized images were analyzed; spot matching across gels and normalization were performed using Delta2D 3.4 (Decodon, Braunschweig, Germany). In order to ensure that the same spot area was quantified in all gels, a master gel was created by fusing all gel images with the maximum intensity option selected in Delta2D. To analyze the significance of protein regulation, a Student's t-test was performed, and statistical significance was assumed for P values less than 0.01.

1.5. In-gel digestion and mass spectrometry analysis

Significantly regulated spots were excised from the gels and tryptic in-gel digestion and peptide extraction were performed as previously described by Dihazi et al. (4). Briefly, gel spots were rinsed twice in 25 mM ammonium bicarbonate (amBic) and once in water, shrunk with 100% acetonitrile (ACN) for 15 min, and dried in a Savant SpeedVac for 20–30 min. All excised spots were incubated with 12.5 ng/μl sequencing grade trypsin (Roche Molecular Biochemicals, Basel, CH) in 25 mM amBic overnight at 37 °C. Peptide extraction was carried out twice using first 50% CAN/1% trifluoroacetic acid (TFA) and then 100% ACN. All extracts were pooled, and the volume was reduced using SpeedVac. Tryptic peptides were dissolved in 0.1% formic acid. One microliter of sample was introduced using a CapLC autosampler (Waters) onto a μ-precolumn TM cartridge, a C18 pepMap (300 μm × 5 mm, 5-μm particle size), and further separated through a C18 pepMap100 nano-SeriesTM (75 μm × 15 cm, 3-

μm particle size) analytical column (LC Packings). The mobile phase consisted of solution A (5% ACN in 0.1% formic acid) and solution B (95% ACN in 0.1% formic acid). The total sample run time was 60 min. The first step consisted of injecting onto a precolumn and washing for 5 min with 0.1% formic acid (30 μl). The washing step was followed by an elution step with an exponential gradient starting with 10% B and ending with 95% B. After 50 min the flow rate was down-regulated by a flow splitter from 5 μl/min pump to 0.25 nl/min. The precolumn was reequilibrated with 0.1% formic acid (30 μl/min) for 5 min. The nanospray needle was held at 2 kV, and the source temperature was held at 40 °C. After chromatographic separation, peptide sequencing was performed on a Q-TOF Ultima Global (Micromass, Manchester, UK) mass spectrometer equipped with a nanoflow ESI Z-spray source in positive ion mode. Multiple charged peptide parent ions were automatically marked and selected in the quadrupole and fragmented in the hexapole collision cell, and their fragment patterns were analyzed by time-of-flight. The data acquisition was performed using MassLynx (Version 4.1) software on a Windows NT PC, while data were further processed on Protein-Lynx-Global-Server (Version 2.5), (Micromass). The raw data files were deconvoluted and deisotoped using the Max EntTM lite algorithm. Processed data were searched against MSDB and Swiss-Prot databases through the Mascot search engine using a peptide mass tolerance of 20 ppm and fragment tolerance of 50 millimass units.

Supplemental Table S2: Glutathione S-transferases, peroxiredoxins and Cytochrome P450 that were significantly regulated in $Calr^{+/-}$ mice kidney.

Identified Proteins	Gene Name	Accession Number	Molecular Weight kDa	Ratio $Calr^{+/-}$ /$Calr^{+/+}$	T-Test (p-value): (p<0.05)
Glutathione S-transferase P 1	Gstp1	GSTP1_MOUSE	24	0.27	0,00014
Glutathione S-transferase Mu 1	Gstm1	GSTM1_MOUSE	26	0.50	< 0,0001
Glutathione S-transferase A3	Gsta3	GSTA3_MOUSE	25	0.25	0,00015
Microsomal glutathione S-transferase 1	Mgst1	MGST1_MOUSE	18	0.007	0,0033
Glutathione S-transferase A4	Gsta4	GSTA4_MOUSE	26	0.48	0,0037
Glutathione S-transferase omega-1	Gsto1	GSTO1_MOUSE	27	0.30	0,014
Glutathione S-transferase Mu 3	Gstm3	GSTM3_MOUSE	26	0.50	0,00043
Glutathione S-transferase Mu 6	Gstm6	GSTM6_MOUSE	26	0.50	0,0057
Peroxiredoxin-2	Prdx2	PRDX2_MOUSE	22	2.02	0.0059
Peroxiredoxin-4	Prdx4	PRDX4_MOUSE	31	201	0.0039
Peroxiredoxin-5	Prdx5	PRDX5_MOUSE	22	6.23	0.0068
Cytochrome P450 2F2	Cyp2f2	CP2F2_MOUSE	56 kDa	0.005	0,00053
Cytochrome P450 2D9	Cyp2d9	CP2D9_MOUSE	57 kDa	0.012	< 0,0001
Cytochrome P450 2E1	Cyp2e1	CP2E1_MOUSE	57 kDa	0.083	0,00077
Cytochrome P450 2C54	Cyp2c54	CP254_MOUSE	56 kDa	0.006	0,00014
Cytochrome P450 2D26	Cyp2d26	CP2DQ_MOUSE	57 kDa	0.010	< 0,0001
Cytochrome P450 3A11	Cyp3a11	CP3AB_MOUSE	58 kDa	0.011	< 0,0001
Cytochrome P450 2C29	Cyp2c29	CP2CT_MOUSE	56 kDa	0.007	< 0,0001
Cytochrome P450 2A12	Cyp2a12	CP2AC_MOUSE	56 kDa	0.007	0,0011
Cytochrome P450 2A5	Cyp2a5	CP2A5_MOUSE	57 kDa	0.338	0,0067
Cytochrome P450 1A2	Cyp1a2	CP1A2_MOUSE	58 kDa	0.014	0,00034
Cytochrome P450 4A12A	Cyp4a12a	CP4CA_MOUSE	58 kDa	0.132	< 0,00010
Cytochrome P450 2C70	Cyp2c70	CP270_MOUSE	56 kDa	0.019	0,00079
Cytochrome P450 2C40	Cyp2c40	CP240_MOUSE	56 kDa	0.029	0,00055
Cytochrome P450 3A13	Cyp3a13	CP3AD_MOUSE	57 kDa	0.053	< 0,0001
Cytochrome P450 4V2	Cyp4v2	CP4V2_MOUSE	61 kDa	0.056	0,00078
Cytochrome P450 4A10	Cyp4a10	CP4AA_MOUSE	58 kDa	0.090	< 0,0001

The Glutathione S-transferases were down- and the peroxiredoxins up-regulated in $Calr^{+/-}$ mice kidney. Cytochrome P450 forms were significantly down-regulated in $Calr^{+/-}$ kidney.

References:

1. Dihazi, H., Asif, A.R., Agarwal, N.K., Doncheva, Y., and Muller, G.A. 2005. Proteomic analysis of cellular response to osmotic stress in thick ascending limb of Henle's loop (TALH) cells. *Mol Cell Proteomics* 4:1445-1458.

2. Gorg, A., Obermaier, C., Boguth, G., Csordas, A., Diaz, J.J., and Madjar, J.J. 1997. Very alkaline immobilized pH gradients for two-dimensional electrophoresis of ribosomal and nuclear proteins. *Electrophoresis* 18:328-337.
3. Bradford, M.M. 1976. A rapid and sensitive method for the quantitation of microgram quantities of protein utilizing the principle of protein-dye binding. *Anal Biochem* 72:248-254.
4. Dihazi, H., Dihazi, G.H., Mueller, C., Lahrichi, L., Asif, A.R., Bibi, A., Eltoweissy, M., Vasko, R., and Mueller, G.A. 2011. Proteomics characterization of cell model with renal fibrosis phenotype: osmotic stress as fibrosis triggering factor. *J Proteomics* 74:304-318.
5. Prakoura N, Politis PK, Ihara Y, Michalak M, Charonis AS. Epithelial calreticulin up-regulation promotes profibrotic responses and tubulointerstitial fibrosis development. Am J Pathol;183:1474-87.

Supplemental Figure 1

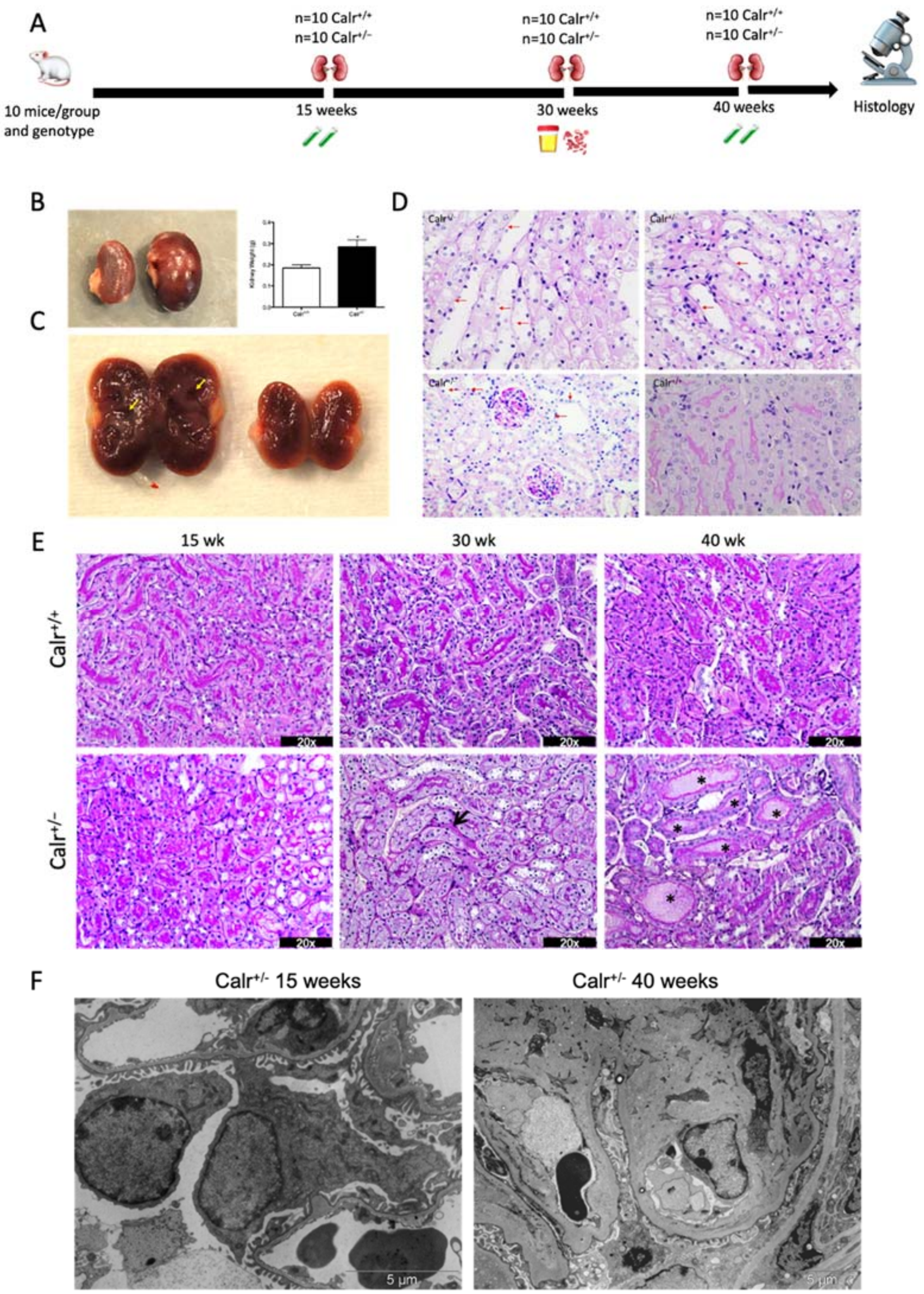

Supplemental Figure S1: Morphometric and histological analysis of kidney from Calr$^{+/+}$ and Calr$^{+/-}$ mice.
A: Illustration of the experimental design for morphological and Immunohistological analysis
B: Gross morphology of kidneys from 40 weeks *Calr*$^{+/+}$ (left) and Calr$^{+/-}$ (right) mice showing an enlargement of *Calr*$^{+/-}$ kidney compared to *Calr*$^{+/+}$. Bar diagram represents the significant increase in *Calr*$^{+/-}$ kidney weight at 40 wk of age. C: Longitudinal sections of kidneys showing progressive internal impairments indicated with arrows (yellow) in *Calr*$^{+/-}$ mice. Genotypes are indicated at the top. wk: weeks. D: Paraffin embedded kidney sections (3 μm) were stained with PAS to compare the kidney structures of *Calr*$^{+/+}$ and *Calr*$^{+/-}$ at 30 wk of age. The PAS staining revealed progressive tubular atrophy (red arrows) in *Calr*$^{+/-}$ kidney, especially the distal convoluted tubule is affected. Glomerular injury is also advanced showing expansion of mesangium with accumulation of PAS-positive material in *Calr*$^{+/-}$ mice (Magnification x20). E: Tubulointerstitial area stained with PAS staining revealed progressive tubulointerstitial damage in *Calr*$^{+/-}$ mice compared to *Calr*$^{+/+}$ mice (Magnification x20). F: Representative electron

micrographs for ultrastructural morphology of kidney section from 15 and 40-weeks-old Calr$^{+/-}$ mice. Images show damaged structures in kidney from 40 weeks old Calr$^{+/-}$ mice compared with younger animals.

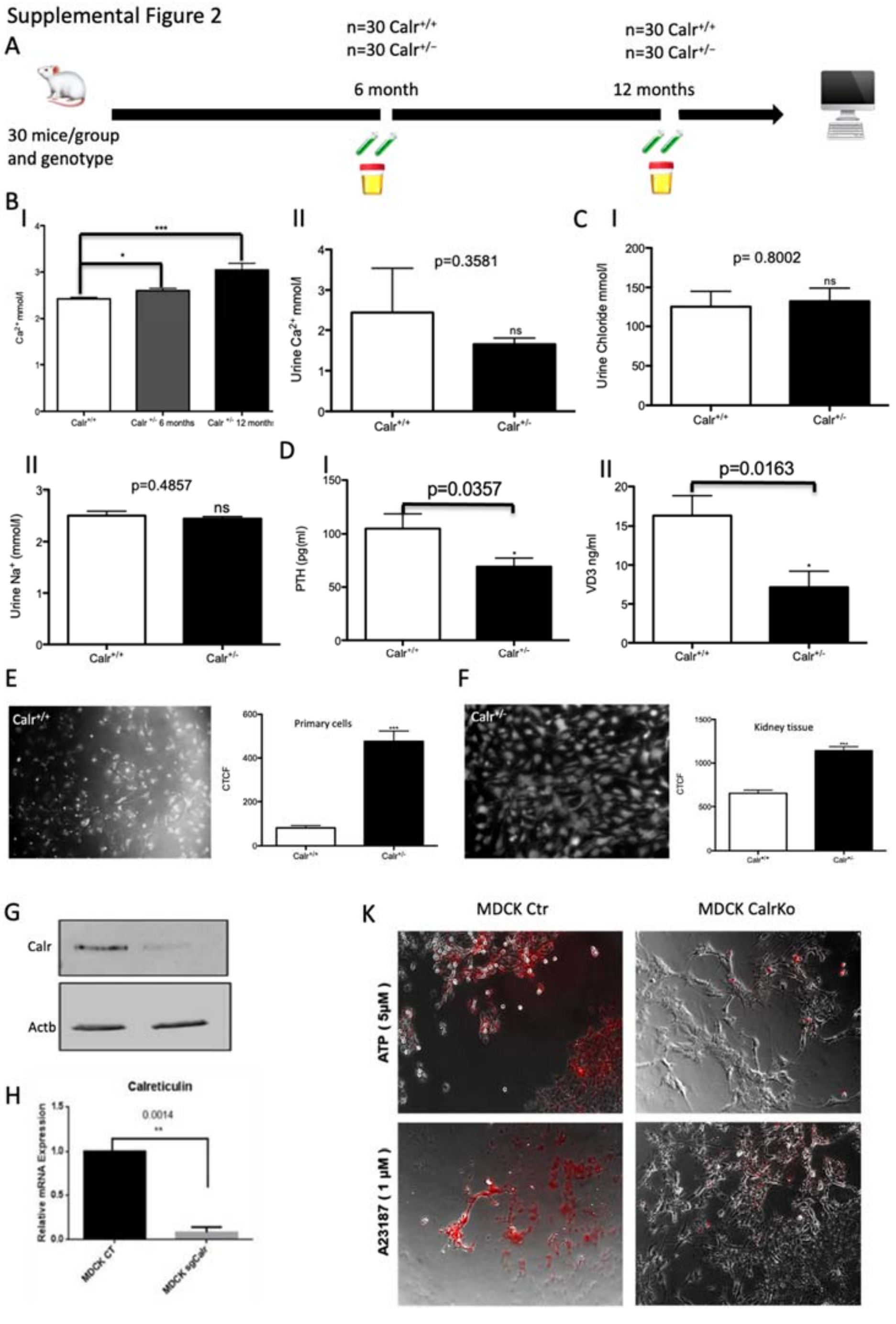

Supplemental Figure S2: Increased calcium concentration and level of calcium handling hormone in *Calr*$^{+/-}$ serum A: Illustration of the experimental design for calcium investigation in serum urine. B: (I), Calcium concentration was estimated in serum of *Calr*$^{+/+}$ and *Calr*$^{+/-}$ mice. The data showed a significant increase in calcium concentration in serum of *Calr*$^{+/-}$ mice, the alteration is age dependent. The calcium level in serum of old *Calr*$^{+/-}$ mice was significantly higher than in serum of *Calr*$^{+/+}$ and young *Calr*$^{+/-}$ mice. (II), Urine calcium concentration in *Calr*$^{+/+}$ and *Calr*$^{+/-}$: no significant difference in calcium excretion could be observed between *Calr*$^{+/+}$ and *Calr*$^{+/-}$ C: Chloride (i) and sodium (II) were estimated in urine samples (n=30 each) using standard routine laboratory methods. No significant difference in chloride and sodium excretion could be measured between Calr$^{+/+}$ and *Calr*$^{+/-}$ samples. D: PTH (I) and VD3 (II) estimations were carried out in serum of *Calr*$^{+/+}$ and *Calr*$^{+/-}$ mice using ELISA. E: Staining of

free calcium in primary cell isolated from $Calr^{+/-}$ and $Calr^{+/+}$ kidney using cell permeant Fura-2. Primary cells from $Calr^{+/-}$ kidney showed increased free Ca^{2+} level than the cells from WT kidney. F: Intact kidney slices were prepared according to Crawford et al. (27), the perfused kidney slices were loaded with Fura-2 at room temperature for 45 min. The $Calr^{+/-}$ tissue showed and increased free calcium level compared to $Calr^{+/+}$. G: Western blot analysis of MDCK protein extract with antibody against Calr showing significant down-regulation in MDCK-CalrKo compared to control. H: qPCR confirmed the significant down-regulation of Calr in MDCK-CalrKo cells. K: Treatment of MDCK-CalrKo and MDCK-controls with ATP and the calcium inophor A23187 confirmed that the Calr-deficiency resulted in impairment of ER-calcium storage and the ability to release calcium to cytosol upon stimulation.

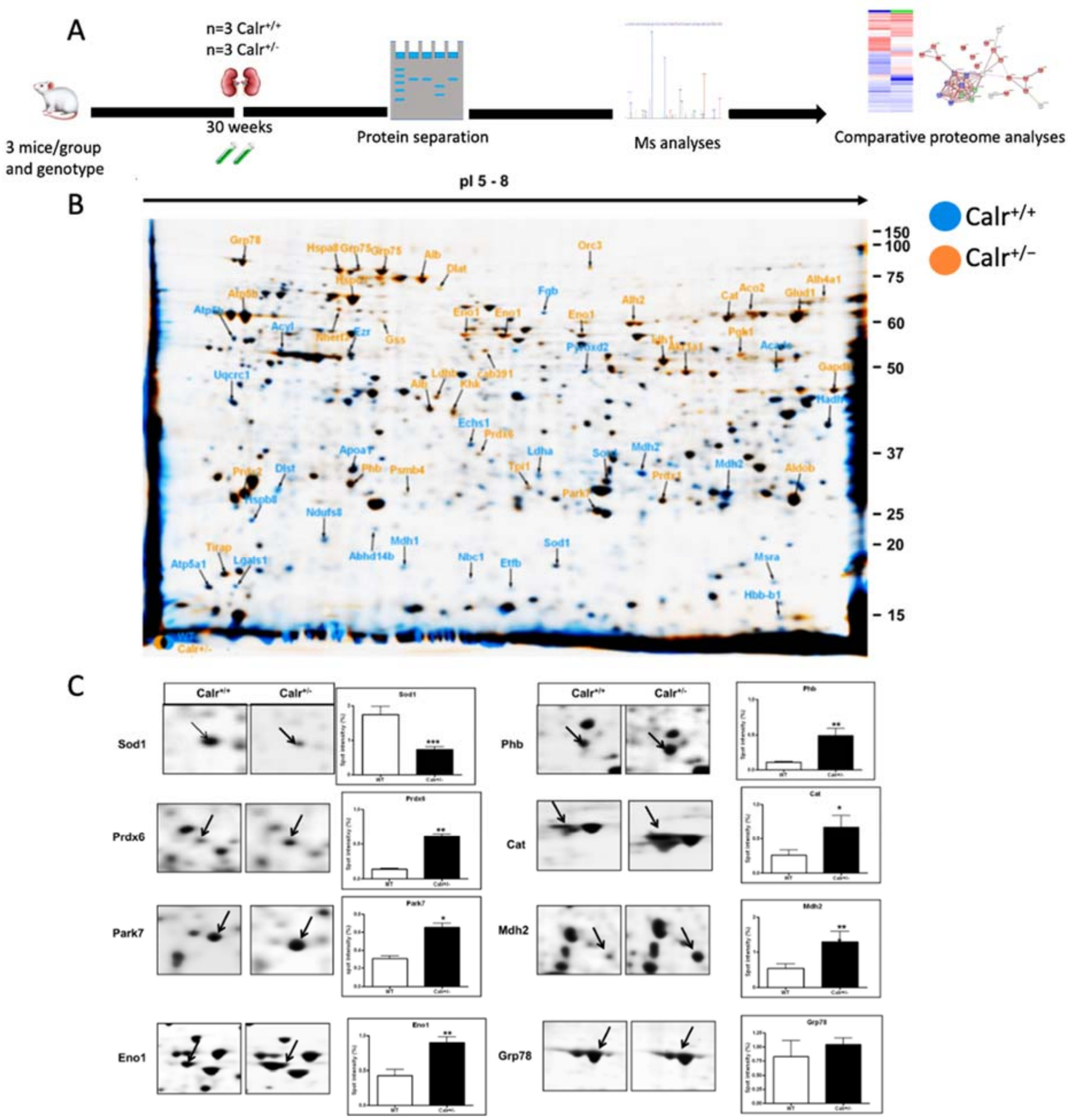

Supplemental Figure S3: 2-DE based comparative proteome analysis of the kidney from $Calr^{+/+}$ and $Calr^{+/-}$ and Gene Ontology (GO) classification of differentially regulated proteins.

A: Illustration of the experimental procedure for the kidney proteome using 2-DE and mass spectrometry. B: Overlapping 2-DE map of $Calr^{+/+}$ and $Calr^{+/-}$ kidneys proteomes. Blue spots indicate higher expression in $Calr^{+/+}$

mice kidney samples compared to Calr$^{+/-}$ mice samples. Orange spots indicate an up-regulation of the proteins in Calr$^{+/-}$. Protein expressed in both phenotypes are overlapping spots and are shown in black. C: Magnified images of regions of interest showing differentially regulated proteins in the Calr$^{+/-}$ mouse kidney. The protein expression quantification for selected proteins is given in form of bar diagrams. Results are given as the means ± SD of the percentage volume of spot from at least three independent experiments ($P < 0.05$).

Supplemental Figure 4

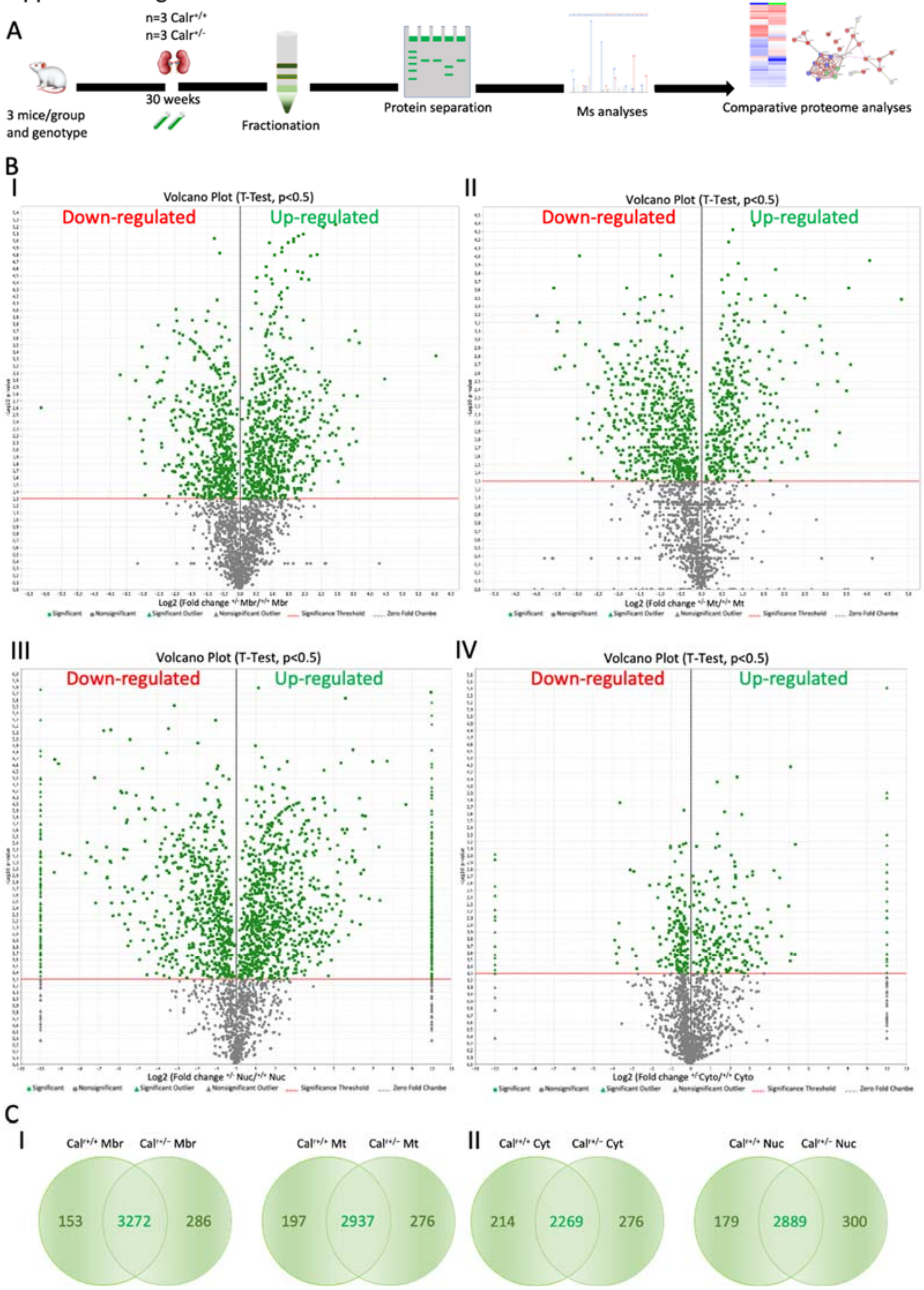

D

I

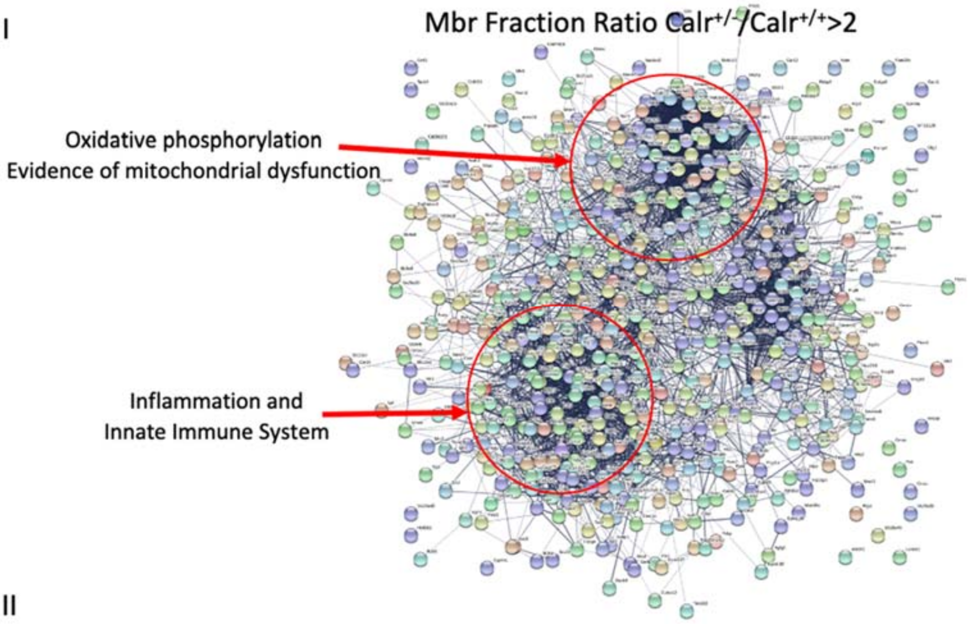
Mbr Fraction Ratio Calr+/-/Calr+/+>2
Oxidative phosphorylation
Evidence of mitochondrial dysfunction
Inflammation and
Innate Immune System

II

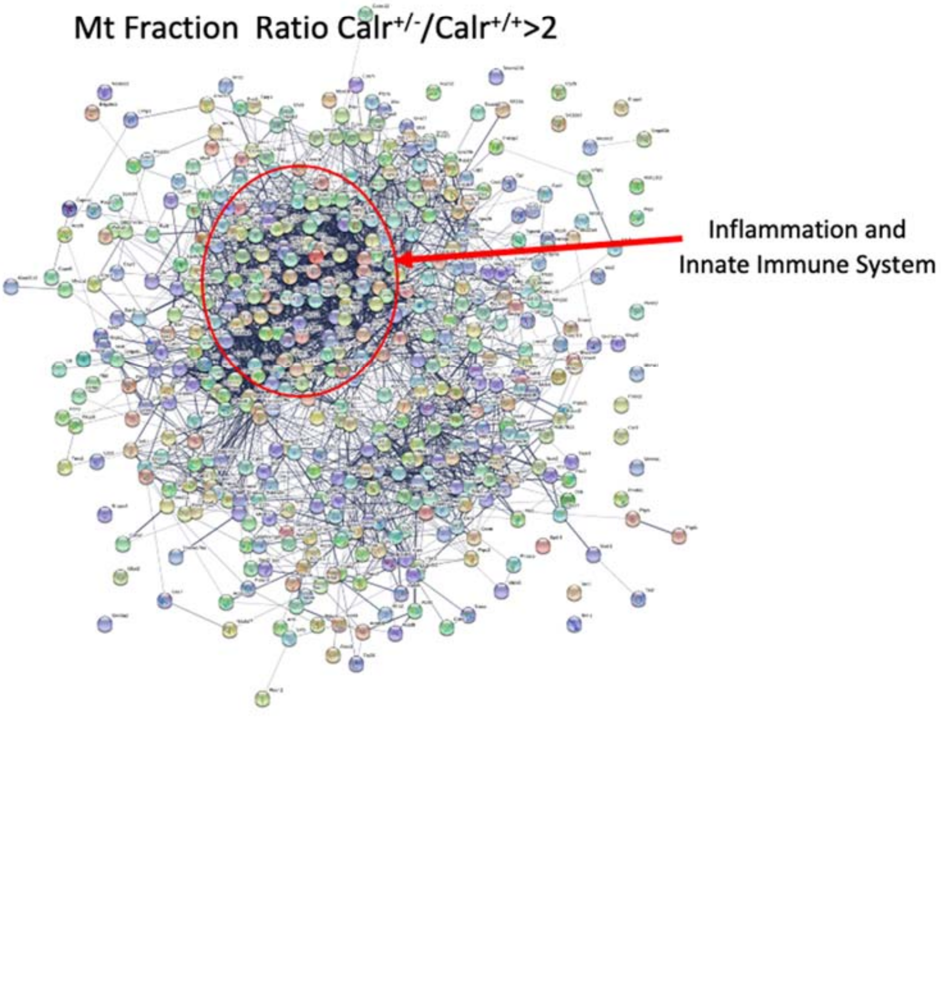
Mt Fraction Ratio Calr+/-/Calr+/+>2
Inflammation and
Innate Immune System

III

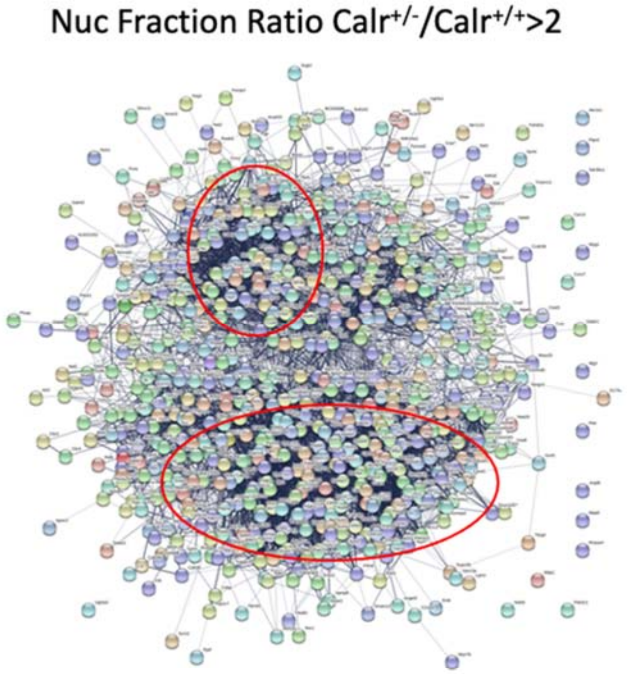

IV

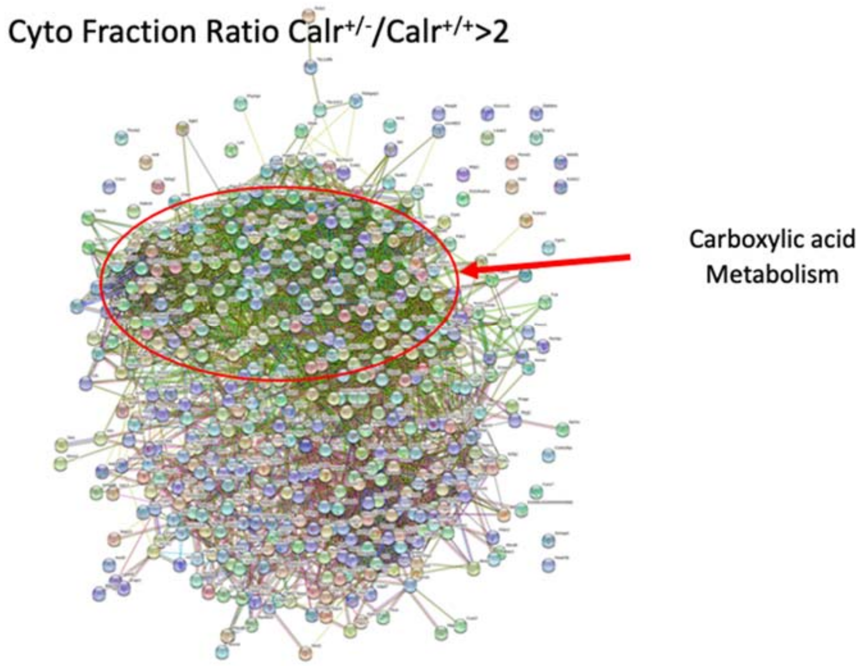

Supplemental Figure S4: Deep analysis of $Calr^{+/+}$ and $Calr^{+/-}$ kidney proteomes

A: The experimental procedure for the deep kidney proteome analysis. Fractionation of the kidney tissue proteome in sub-proteomes resulted in 4 fractions: Membrane, mitochondrial, cytosolic and nuclear. The fraction proteomes were analyzed combining 1DE and mass spectrometry B I-IV: Volcano plot analysis of the identified and quantified proteins allowed the easy identification of proteins differentially expressed between $Calr^{+/+}$ and $Calr^{+/-}$ kidney. C I, II: Pie diagram presentation of the protein identified in the different fractions. The Pie diagram allow to identify proteins expressed only in one phenotype or the overlapping proteins which were identified in both phenotypes. D I-IV: Protein interaction networks between the proteins found to be up-regulated in $Calr^{+/-}$ mice kidney were generated using the protein networks software String (https://string-db.org).

Supplemental Figure 5

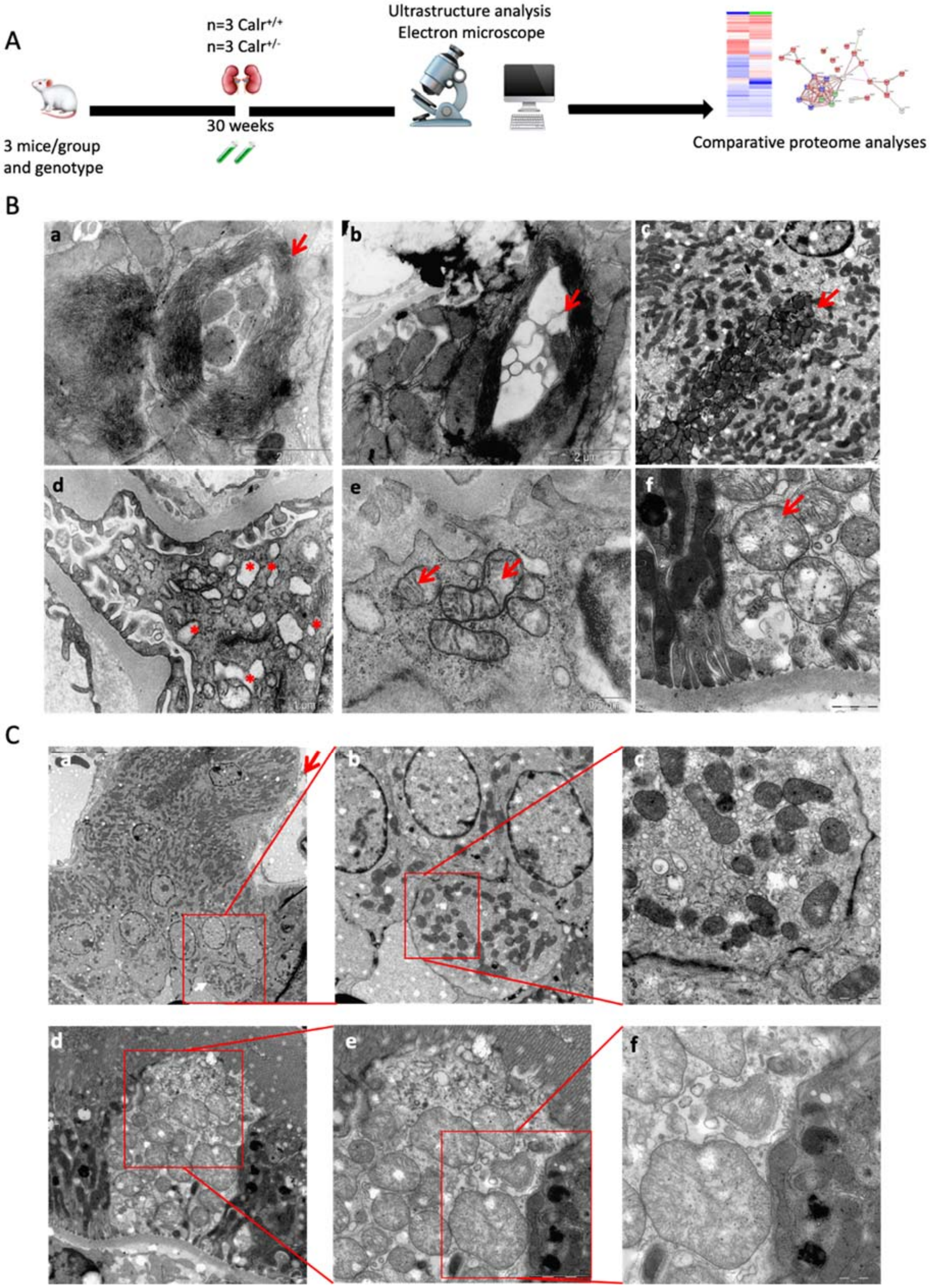

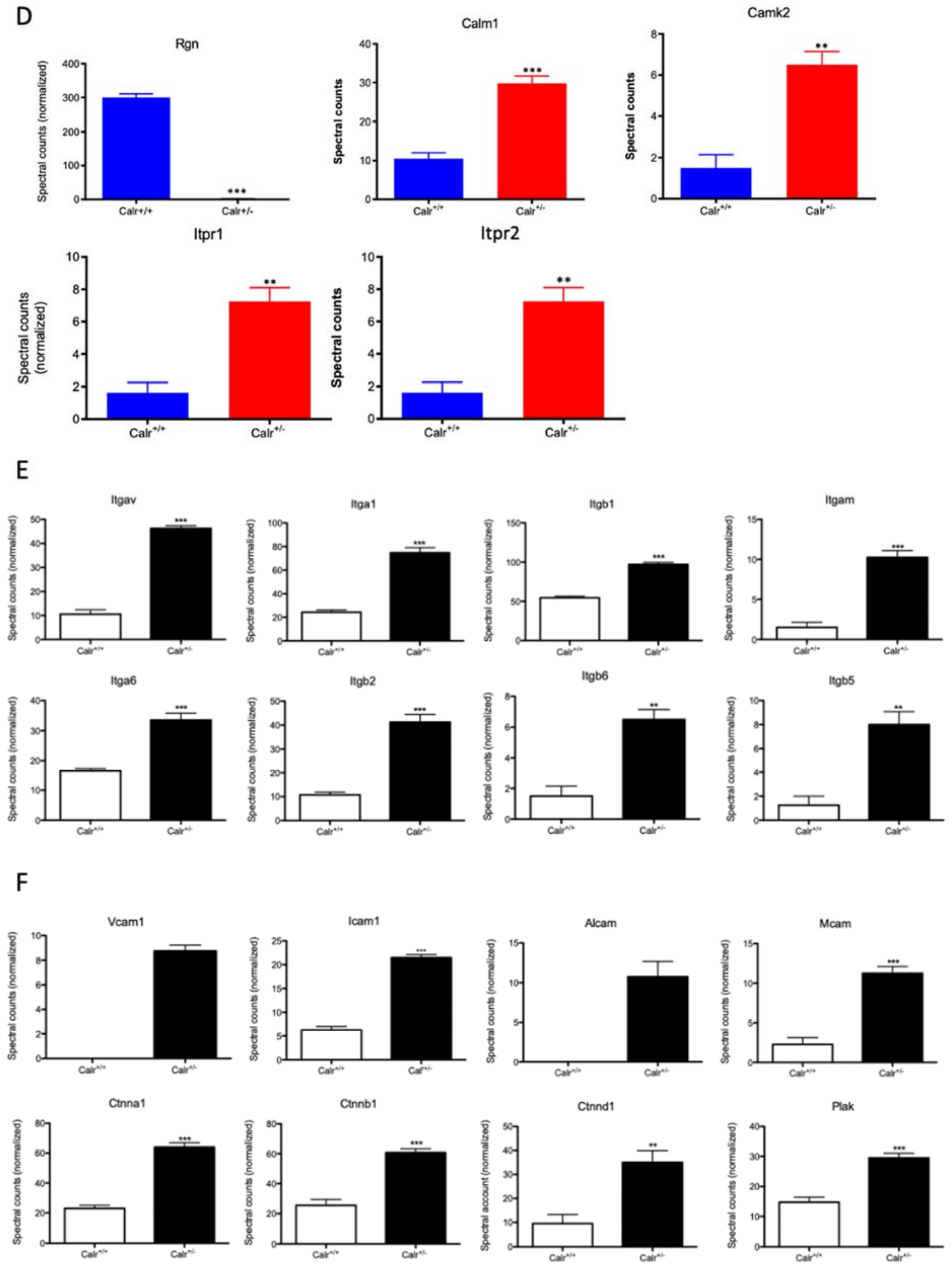

Supplemental Figure S5: Ultrastructural analysis of kidney tissue demonstrating intensive autophagy in Calr$^{+/-}$ kidney A: Illustration of the experimental design for Ultrastructure analysis and comparative proteome investigation. B: Representative electron micrographs for ultrastructural morphology of mitochondria from Calr$^{+/-}$ kidney. Tubular cells with autophagosomes containing mitochondria **(a),** or mitochondria in advanced stage of

autophagy a number of mitochondria are enclosed in a membranous network in tubular cell, a progressive autophagosome with completely damaged mitochondria **(b)**, Robust number of mitochondria in certain tubular cells **(c)**. Podocyte with damaged vacuolated mitochondria highlighted with red asterisks **(d),** higher magnification image of a podocyte illustrating mitochondrial swelling and damage with disordered cristae indicated with red arrows **(e),** Mitochondrial swelling in a tubular cell indicated with arrow **(f). C:** Autophagosome with mitochondria **(a, b, c)**, accumulation of swelling mitochondria in large membrane structure **(d, e, f)**. D: Analysis of proteomic data showed a down-regulation of regucalcin, an inhibitor of Camk2 and PKC, while Calm-1 and Camk2 were up-regulated. E, F: Quantification of proteomics data demonstrating an up-regulation in the expression the key proteins involved in the transcellular plasma cell infiltration in $Calr^{+/-}$ kidney.
